# A bioengineered platform reveals progressive impairment of skeletal muscles by metabolic dysfunction-associated steatotic liver disease-derived factors

**DOI:** 10.1242/dmm.052820

**Published:** 2026-09-17

**Authors:** Armando Cortés-Reséndiz, Juan M. Fernández-Costa, Maria Sabater-Arcís, Francesco De Chiara, Javier Ramón-Azcón

**Affiliations:** ^1^ Institute for Bioengineering of Catalonia (IBEC), 08028 Barcelona, Spain; ^2^ Universitat Autónoma de Barcelona (UAB), 08193 Barcelona, Spain; ^3^ Neuromuscular Diseases Research Group, Institut de Recerca Sant Pau (IR SANT PAU), Hospital de la Santa Creu i Sant Pau, 08041 Barcelona, Spain; ^4^ CIBER de Enfermedades Raras, Instituto de Salud Carlos III, 3-5 28029 Madrid, Spain; ^5^ Institució Catalana de Recerca i Estudis Avançats (ICREA), Passeig de Lluís Companys, 23, E08010 Barcelona, Spain

**Keywords:** MASLD, Skeletal muscle wasting, 3D bioengineered tissue, Liver–muscle crosstalk, Metabolic dysfunction-associated steatohepatitis

## Abstract

Metabolic dysfunction-associated steatotic liver disease (MASLD) affects >30% of the global population and is increasing in prevalence to become the most common liver disorder. MASLD can influence distal organs via secreted mediators, and up to 43% of patients show sarcopenia or related muscle-wasting phenotypes. We developed a modular human three-dimensional bioengineered liver–muscle platform to dissect causal liver–muscle crosstalk. Liver constructs recapitulated steatosis and transition to metabolic dysfunction-associated steatohepatitis (MASH), with inflammatory, apoptotic, immune cell-recruiting and pro-fibrotic signatures. Transferring liver-derived conditioned media to engineered muscles revealed severity-dependent impairment of contractile performance, altered protein balance and myogenic regulation. Contractility declined progressively from steatosis to MASH. Steatotic liver conditioned media reduced myotube size, whereas MASH conditioned media triggered a compensatory program that partially preserved size despite functional loss. Muscle exposure to MASLD mediators was associated with disrupted autophagic degradation, consistent with impaired flux and accumulation of autophagosomes and lysosomes. This scalable human platform enables systematic investigation of MASLD extrahepatic mechanisms and provides a testbed for perturbations and interventions targeting liver-driven muscle wasting.

## Teaser

**Summary:** Bioengineered human liver–muscle constructs reveal that metabolic dysfunction-associated steatotic liver disease/metabolic dysfunction-associated steatohepatitis-derived secreted factors are sufficient to drive severity-dependent muscle dysfunction via altered contractility, proteostasis and autophagy.

## INTRODUCTION

Metabolic dysfunction-associated steatotic liver disease (MASLD) is a multi-system disease spectrum affecting >30% of the global population, with projections suggesting that prevalence could reach 55.4% by 2040 (Younossi et al., 2023, 2025). Beyond its increasing incidence – including in children and adolescents – MASLD carries a growing health burden, with 6.6% of patients developing liver cancer (Younossi et al., 2025). Although the hepatic disease course is well defined – from triglyceride accumulation in hepatocytes (steatosis) to metabolic dysfunction-associated steatohepatitis (MASH), marked by hepatocellular injury, lobular inflammation, cell death and progressive extracellular matrix (ECM) remodeling driven in part by non-parenchymal cells – MASLD is tightly linked to systemic metabolic comorbidities such as obesity, insulin resistance and elevated risk of type 2 diabetes (Ballestri et al., 2016; Li et al., 2022, 2024; Chan et al., 2023; Tan et al, 2023; Bril et al., 2024; Do et al., 2025).

MASLD has extrahepatic effects on whole-body glucose and lipid homeostasis, underscoring the liver's role as an endocrine and inflammatory hub (Ferrara et al., 2019; Sandireddy et al., 2024). The liver can influence distal organs through secreted mediators, including hepatokines, with skeletal muscle emerging as a key target given its central contributions to metabolic regulation (Kang et al., 2020; Montgomery et al., 2023; Fan et al., 2025). Clinically, muscle impairment is increasingly recognized across the MASLD spectrum and is associated with worse outcomes (Bhanji et al., 2017a,b; Feng et al., 2024). Although sarcopenia is classically defined as a progressive loss of skeletal muscle mass, strength and function (Cruz-Jentoft et al., 2019) and is well documented in advanced liver disease, reports indicate that up to 43% of patients with MASLD exhibit sarcopenia or related phenotypes (Montano-Loza et al., 2016; Kang and Yoon, 2023). Here, we refer to these manifestations as muscle wasting (MW) to emphasize their etiological link to a diseased liver milieu rather than aging, aligning with an emerging view that disrupted inter-organ communication – particularly within the liver–muscle axis – contributes to muscle deterioration in metabolic liver disease (Tandon et al., 2016; Bhanji et al., 2018; Linge et al., 2021).

Despite these advances, the mechanistic basis of liver–muscle crosstalk in MASLD remains incompletely understood (Bhanji et al., 2017a; Viswanath et al., 2024). A major barrier is the lack of human-relevant experimental systems that faithfully reproduce key tissue phenotypes while enabling controlled interrogation of organ–organ communication (De Chiara et al., 2024; Fernández-Costa et al., 2024). As a result, existing approaches remain inadequate for dissecting causal roles of liver-derived factors in driving muscle dysfunction, motivating the development of inter-organ models that can capture and test clinically relevant secreted mediators in a tractable manner. Traditional two-dimensional (2D) monocultures are scalable and experimentally convenient, but they poorly reproduce multicellular interactions, ECM remodeling and paracrine signaling that shape MASLD progression, and they often underestimate the biological impact of secreted factors. Animal models capture systemic physiology but introduce species-specific differences and make it difficult to isolate organ-intrinsic mechanisms or attribute distal phenotypes to defined liver-derived cues. In contrast, three-dimensional (3D) bioengineered tissue platforms provide controllable, human-relevant systems that better preserve differentiated phenotypes and cell–cell communication, while enabling collection of conditioned media (CM) for downstream profiling and functional testing – an essential feature for mechanistic dissection of secreted mediators in modular inter-organ designs (Mughal et al., 2022).

Within this framework, HepaRG/LX-2 co-cultures capture hepatocyte–stellate cell crosstalk central to MASLD progression to MASH, and 3D constructs can support hepatocyte differentiation while limiting artefactual hepatic stellate cell (HSC) activation driven by non-physiological substrate stiffness. To model key pathogenic inputs, we applied a two-hit strategy combining non-esterified fatty acids (NEFAs) and lipopolysaccharide (LPS) to mimic lipotoxicity and inflammatory stress, then used CM transfer to the muscle tissues to probe causality. Complementarily, 3D skeletal muscle models coupled to electrical pulse stimulation (EPS) enable functional readouts that extend beyond viability or histology alone (Tejedera-Villafranca et al., 2023; Mughal et al., 2025; Ruiz-Gutiérrez et al., 2025). Building on these capabilities, we set out to (1) engineer 3D liver constructs that recapitulate stage-specific MASLD features spanning steatosis to MASH-like phenotypes, (2) leverage our established functional 3D skeletal muscle platform for quantitative contractility assessment, and (3) demonstrate that liver-derived secreted factors are sufficient to drive muscle dysfunction, structural remodeling, altered gene expression and protein-balance disruption consistent with clinical MASLD-associated MW. By design, this modular approach isolates MASLD-derived signals from clinical confounders, enabling the identification of mechanistic mediators and providing a robust platform to evaluate intervention strategies targeting liver–muscle crosstalk in MASLD-associated MW in accordance with the 3Rs (replacement, reduction and refinement) principle.

## RESULTS

### Two-hit treatment induces progressive steatosis and hepatocellular injury in HepaRG/LX-2 constructs

We selected HepaRG cells for their retained hepatocyte-like phenotype and responsiveness to lipotoxic stimuli. Metabolic and lysosomal activity were assessed using 3-(4,5-dimethylthiazol-2-yl)-5-(3-carboxymethoxyphenyl)-2-(4-sulfophenyl)-2H-tetrazolium (MTS) and Neutral Red (NR) assays, respectively. Initial 2D dose–response experiments established 400 μM non-esterified fatty acids (NEFAs) as the optimal concentration, inducing significant lipid accumulation and metabolic changes without excessive toxicity, as shown by increased metabolic activity measured with the MTS assay (+81%, *P*=0.03). This concentration did not significantly increase lysosomal activity measured with the NR assay (+34.6%, *P*=0.2), but yielded robust Oil Red O staining, while lower concentrations (≤40 μM NEFAs) showed no effect (Fig. S1). LX-2 cells are well-characterized immortalized human HSCs that retain key features of cytokine signaling, retinoid metabolism and fibrogenesis (Xu et al., 2005; Arriola Benitez et al., 2013; Weiskirchen et al., 2013; Feng et al., 2014).

To model MASLD in three dimensions, liver constructs were fabricated by encapsulating HepaRG and LX-2 cells at a 2:1 ratio in collagen-based hydrogels (Fig. 1A). Constructs remained structurally stable with homogeneous cell distribution (Fig. 1B). Albumin-positive hepatocytes were present at baseline (day 0; Fig. S2A) and in vehicle constructs at day 7 (Fig. S2B), confirming the preservation of hepatocyte phenotype. The disease progression was approached by treating the 3D livers with a first hit of 400 μM NEFAs, as established previously. At day 4, a second hit was performed by adding a co-treatment group, which received 400 μM NEFAs plus 0.1 μg ml^−1^ LPS (NEFAs+LPS), a potent stellate cell activator (Fig. 1A).

**Fig. 1.**
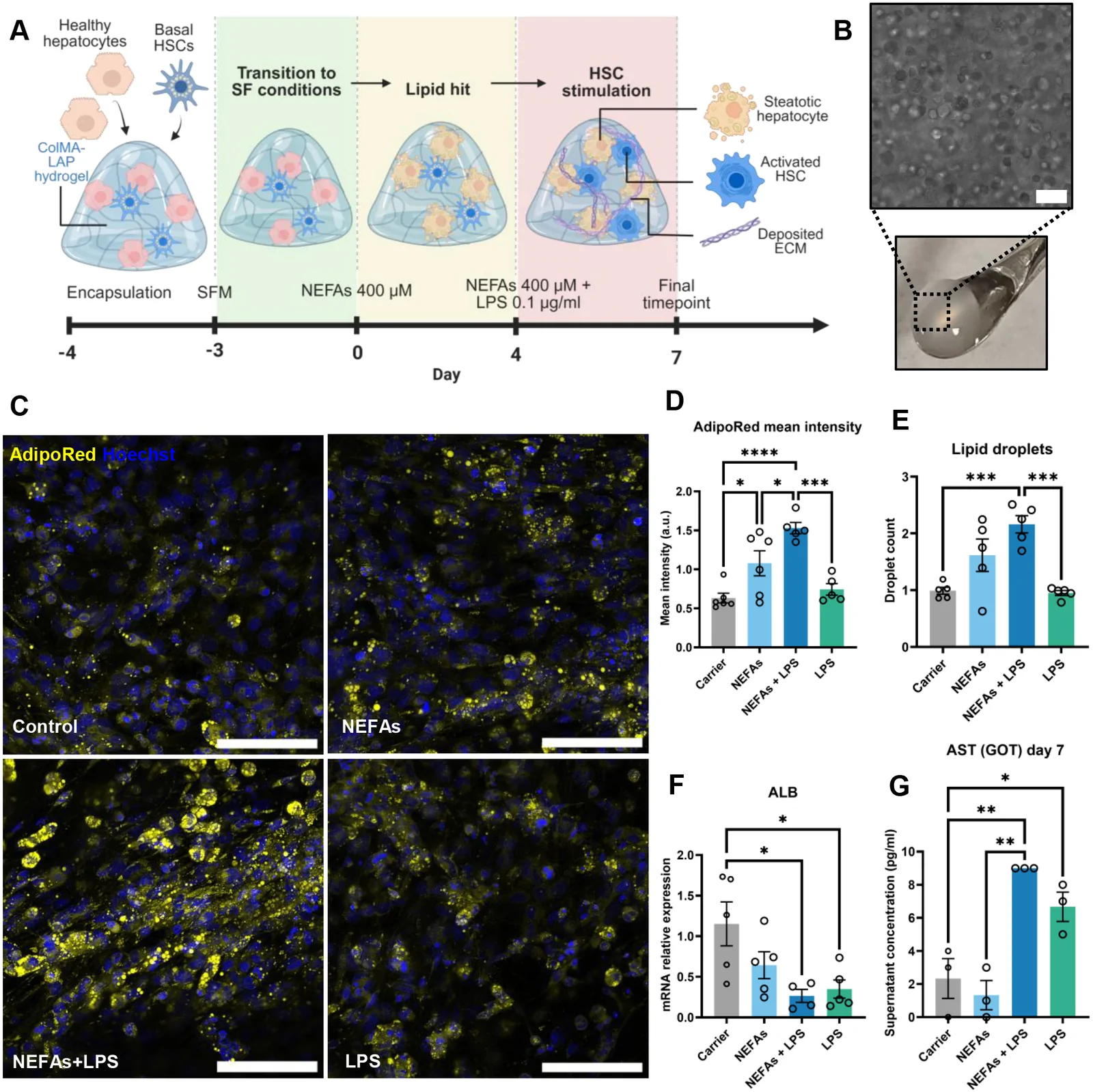
**Generation and characterization of a 3D HepaRG/LX-2 liver model of metabolic dysfunction-associated steatotic liver disease progression.** (A) Schematic representation of construct fabrication and experimental timeline of the model. ColMA, methacrylated collagen; ECM, extracellular matrix; HSC, hepatic stellate cell; LAP, lithium phenyl(2,4,6-trimethylbenzoyl)phosphonate; LPS, lipopolysaccharide; NEFA, non-esterified fatty acid; SF, serum-free; SFM, serum-free medium. (B) Representative brightfield image showing cell distribution within the 3D liver construct at the final timepoint. Scale bar: 50 μm. (C) Representative confocal *z*-projections of live constructs stained with AdipoRed™ at the final timepoint. Nuclei were stained with Hoechst 33342. Scale bars: 100 μm. (D) Quantification of AdipoRed™ mean fluorescence intensity normalized to Hoechst 33342 signal. a.u., arbitrary units. (E) Lipid droplet count normalized to nuclei number. (F) Relative mRNA expression of albumin (*ALB*) at the final time point, using the carrier condition as the reference group and β-actin (*ACTB*) as the housekeeping gene. Expression levels were calculated by the ΔΔCt method. (G) Supernatant analysis of aspartate aminotransferase (AST) levels at the final timepoint. Results are expressed as mean±s.e.m. and were compared using one-way analysis of variance (ANOVA) followed by Tukey's post hoc tests when appropriate. *P<0.05; **P<0.01; ***P<0.001; ****P<0.0001.

Lipid accumulation at day 7 was assessed by AdipoRed™ staining in live constructs (Fig. 1C). Treatment with NEFAs significantly increased AdipoRed mean intensity (+70.6%, *P*=0.027) and lipid droplet area (+479.4%, *P*=0.01), although droplet count changes only trended upward (+63.1%, *P*=0.07). Co-treatment with NEFAs+LPS further exacerbated steatosis across all metrics: AdipoRed intensity (+141.7%, *P*<0.0001), droplet count (+118.1%, *P*=0.0007) and droplet area (+506.5%, *P*=0.007). The synergistic effect was confirmed by significant differences between NEFAs and NEFAs+LPS groups (*P*=0.035 for intensity). Control and LPS-alone constructs showed only baseline lipid content, confirming that steatosis required lipid loading.

Albumin expression, a marker of hepatocyte function, was impaired in treated groups (Fig. 1F). NEFAs alone caused a non-significant change in the mean expression (−44.11%, *P*=0.22), whereas co-treatment with NEFAs+LPS resulted in severe reduction (−76.9%, *P*=0.02). Notably, LPS alone also significantly decreased albumin expression (−69.6%, *P*=0.02), indicating that inflammatory stimulation can impair hepatocyte function independently of steatosis. Immunofluorescence confirmed these findings, with fewer albumin-positive cells in LPS-containing groups. Vimentin staining revealed activated stellate cell networks in treated constructs, most prominently in the NEFAs+LPS group (Fig. S2B). Aspartate aminotransferase (AST) is a classical indicator of hepatocellular damage. AST activity in supernatants was significantly elevated in NEFAs+LPS constructs (9 U L^−1^; +285.7%, *P*=0.002), with a significant change in the LPS-treated groups (+185.7%, *P*=0.03) (Fig. 1G).

To assess hepatocellular damage, we measured AST release and cell viability. AST activity in culture supernatants was significantly elevated in both LPS-containing groups (LPS alone, +185.7%, *P*=0.03; NEFAs+LPS, +285.7%, *P*=0.002) (Fig. 1G), with the co-treatment showing the most severe injury. Consistent with these findings, LIVE/DEAD staining revealed that treatments with NEFAs and NEFAs+LPS reduced viability by 15.9% and 22.9% (*P*=0.03), respectively, while treatment with LPS alone caused a non-significant 14.9% reduction in viability (Fig. 2E,F). Together, these results demonstrate that the two-hit model induces progressive hepatocellular injury beyond simple steatosis.

**Fig. 2.**
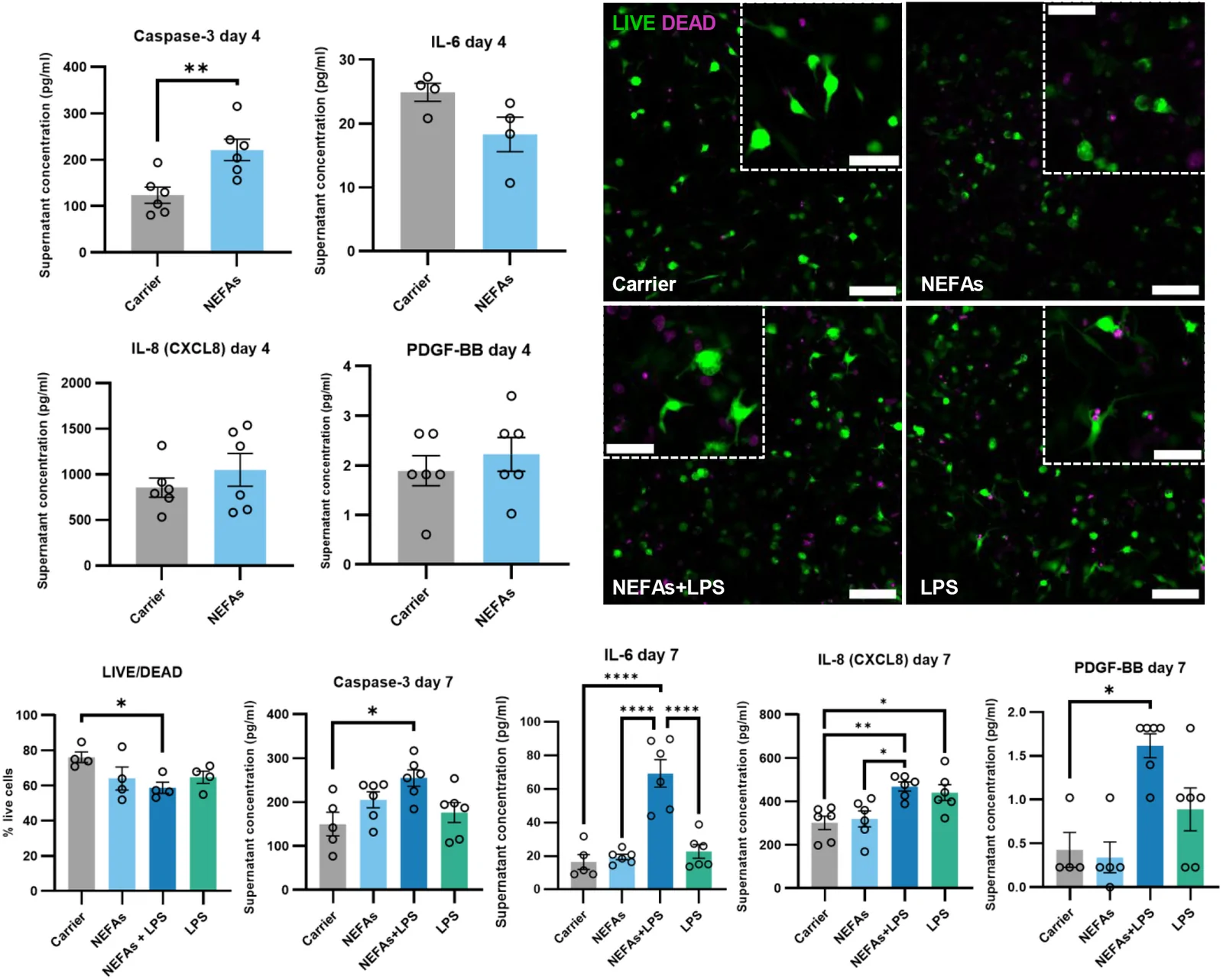
**Markers of cell viability, apoptosis and inflammation.** (A-D) Analysis of capsase-3 (A), IL-6 (B), IL-8 (C) and PDGF-BB (D) in supernatants at day 4 (after lipid hit). (E) Representative confocal images of LIVE/DEAD™ staining at the final timepoint. Scale bars: 100 μm; 50 μm (insets). (F) Percentage of live cells over total cells from LIVE/DEAD™ staining. (G-J) Analysis of capsase-3 (G), IL-6 (H), IL-8 (I) and PDGF-BB (J) in supernatants at day 7 (after lipid hit and hepatic stellate cell stimulation). Results are expressed as mean±s.e.m. Groups at day 4 were compared with unpaired tests with Welch's correction. Groups at day 7 were compared using one-way ANOVA followed by Tukey's, Dunnett's or Kruskal–Wallis post hoc tests when appropriate. *P*<0.05; **P<0.01; ****P<0.0001.

### Co-treatment with NEFAs and LPS synergistically activates inflammatory and apoptotic pathways

To assess progression from steatosis to MASH-like stages, we measured apoptotic and inflammatory markers at days 4 and 7 post-treatment. Caspase-3, a central apoptotic effector, showed rapid elevation at day 4 in NEFA-treated constructs (+79.5%, *P*=0.007). This elevation under NEFAs was only apparent by day 7, as it did not reach significance (+37.1%, *P*=0.3) (Fig. 2A,G). Co-treatment with NEFAs+LPS maintained significantly elevated caspase-3 on day 7 (+70.1%, *P*=0.01). IL-6 showed minimal changes on day 4 across all groups (Fig. 2B). By day 7, however, a marked inflammatory response emerged: treatment with NEFAs alone did not yield differences in IL-6 levels (+18.24%, *P*=0.9), whereas co-treatment with NEFAs+LPS produced a >3-fold increase (+323.2%, *P*<0.0001), significantly exceeding levels in both the NEFAs- and LPS-alone groups (Fig. 2H), indicating strong synergy between lipotoxicity and HSC stimulation. Similarly, IL-8 expression did not change significantly on day 4 but reached significance on day 7 in NEFAs-alone (+55.8%, *P*=0.007) and LPS-alone (+46.5%, *P*=0.03) groups (Fig. 2D,I). PDGF-BB, which promotes HSC proliferation and fibrogenesis, showed no significant changes on day 4. On day 7, only co-treatment with NEFAs+LPS significantly upregulated PDGF-BB (+279%, *P*=0.02) (Fig. 2J), suggesting activation of pro-fibrotic pathways specifically under combined metabolic and inflammatory stress.

### HepaRG/LX-2 constructs exhibit progressive fibrotic remodeling under two-hit conditions

Given evidence of HSC activation, we investigated ECM remodeling through histological, biochemical and molecular approaches. Picrosirius Red staining revealed collagen deposition forming pericellular halos around cell clusters (Fig. 3A). Polarized light microscopy identified both type I collagen (yellow/orange birefringence) and type III collagen (green birefringence) fibers extending between clusters, consistent with early fibrotic remodeling and bridging fibrosis patterns (Fig. 3B).

**Fig. 3.**
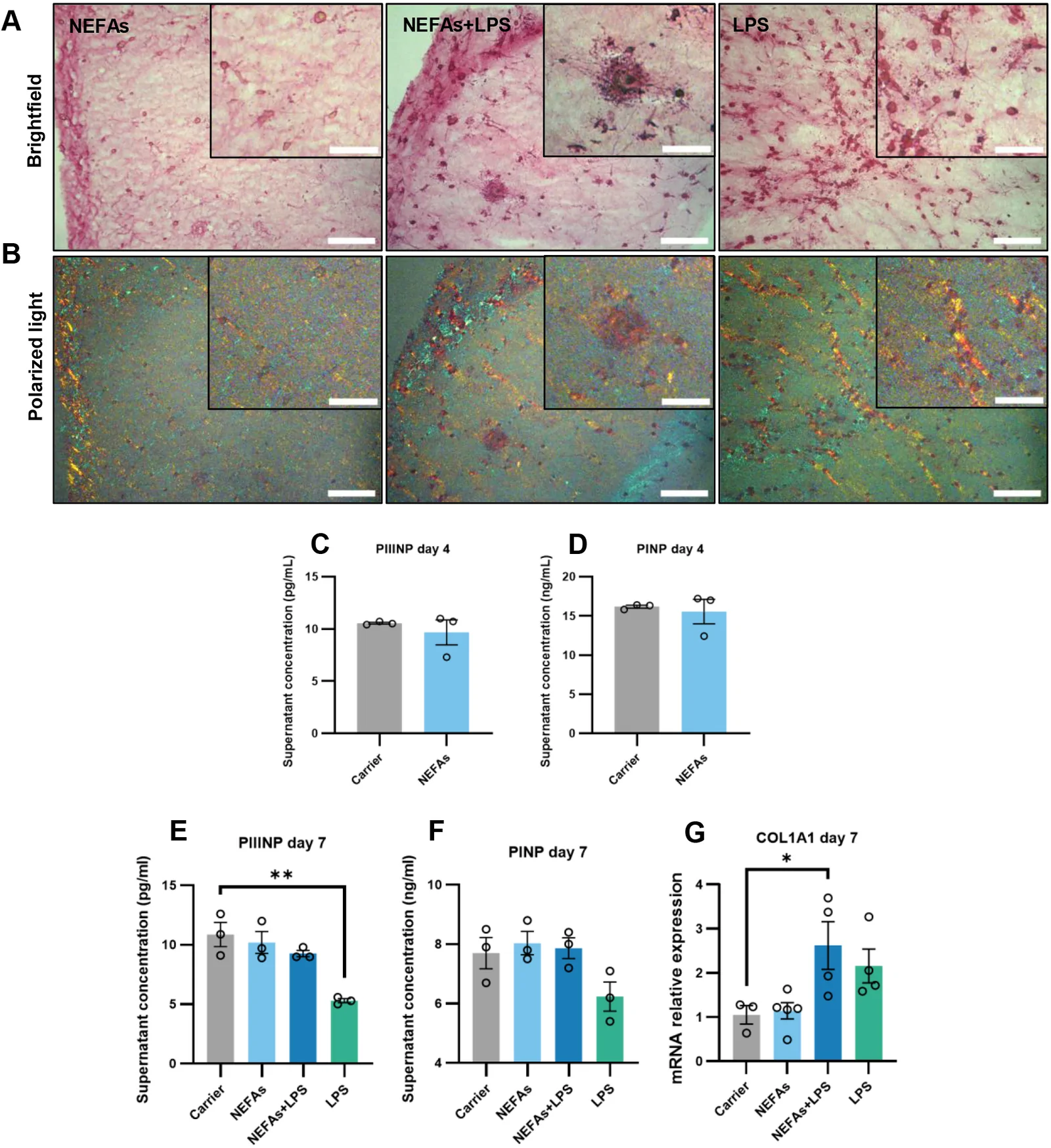
**Histological characterization of liver construct cryosections and supernatant analysis at day 4 (after lipid hit) and 7 (after lipid hit and hepatic stellate cell stimulation).** (A) Representative brightfield images of liver construct cryosections stained with Picrosirius Red, with magnified insets highlighting collagen-rich regions. Collagen fibers are shown in red. Scale bars: 100 μm; 50 μm (insets). (B) Polarized-light microscopy of the same tissue sections shown in A, with magnified insets highlighting birefringent collagen signal. Yellow/orange birefringence is consistent with thicker collagen fibers, whereas green birefringence is consistent with thinner collagen fibers. Scale bars: 100 μm. (C-F) Collagen type-III and type-I propeptides [procollagen type III N-terminal propeptide (PIIINP) at day 4 (C), procollagen type I N-terminal propeptide (PINP) at day 4 (D), PIIINP at day 7 (E) and PINP at day 7] in supernatant. (G) Relative mRNA expression of collagen type I alpha 1 chain (*COL1A1*) at the final timepoint, using the carrier condition as the reference group and β-actin (*ACTB*) as the housekeeping gene. Expression levels were calculated by the ΔΔCt method. Results are expressed as mean±s.e.m. Groups at day 4 were compared with unpaired tests with Welch's correction. Groups at day 7 were compared using one-way ANOVA followed by Tukey's or Dunnett's post hoc tests when appropriate. *P<0.05; **P<0.01.

To quantify collagen turnover, we measured procollagen type I N-terminal propeptide (PINP) and procollagen type III N-terminal propeptide (PIIINP) in culture supernatants. At day 4, neither propeptide showed significant changes (Fig. 3C,D). At day 7, PIIINP levels did not show significant differences under treatments with NEFAs (−6.1%, *P*=0.8) or NEFAs+LPS (−14.7%, *P*=0.3), whereas treatment with LPS alone significantly reduced PIIINP levels (−51.2%, *P*=0.001) (Fig. 3E). PINP showed non-significant upward shifts under treatments with NEFAs and NEFAs+LPS (Fig. 3F). The lack of elevated propeptides in supernatants may reflect either propeptide retention within the dense 3D matrix or a shift in collagen dynamics not captured by these circulating markers.

At the transcriptional level, *COL1A1* expression was significantly upregulated in NEFAs+LPS constructs at day 7 (+149%, *P*=0.03), indicating active collagen synthesis and ECM remodeling specifically under combined metabolic and inflammatory stress. Together, the histological collagen staining, birefringent collagen signal, *COL1A1* upregulation and collagen propeptide measurements support ECM remodeling and fibrosis-associated changes under combined metabolic and inflammatory stress.

### CM from MASH-like constructs impair skeletal muscle function

To investigate whether secreted factors from diseased livers could directly affect skeletal muscle, we generated 3D skeletal muscle constructs and exposed them to CM from our liver models for 48 h (Fig. 4A). CM from steatotic liver (SL) constructs and MASH-like liver constructs are hereafter referred to as SL-CM (SL-treated) and MASH-CM (MASH-treated), respectively. Immortalized human myoblasts were encapsulated in Matrigel^®^-fibrin hydrogels and cultured for 16 days to achieve full differentiation (Tejedera-Villafranca et al., 2023; Mughal et al., 2024). By day 16, constructs displayed mature myotube phenotypes with aligned, multinucleated fibers throughout, as confirmed by confocal microscopy (Fig. S3A,B).

**Fig. 4.**
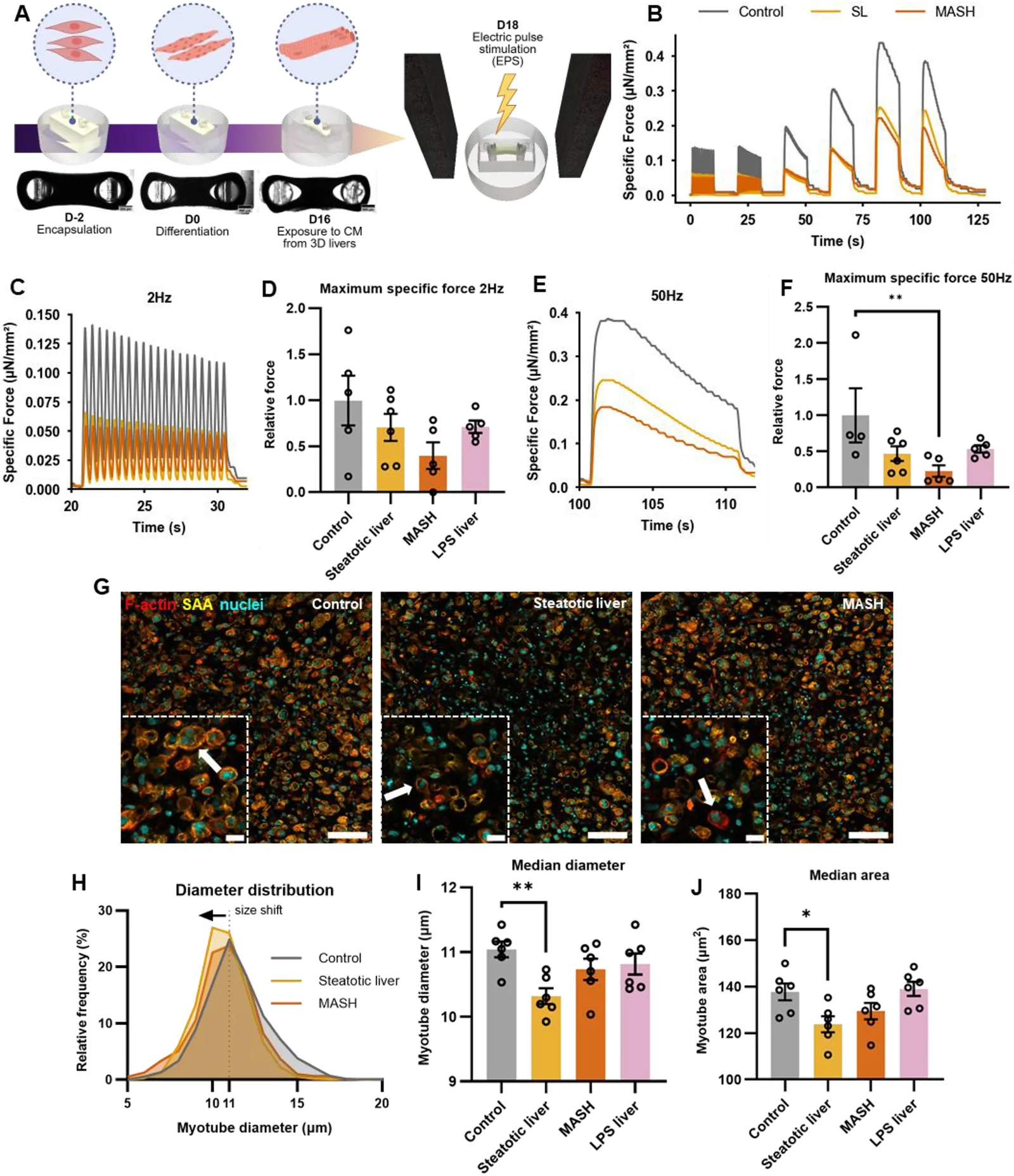
**Functional and morphometric evaluation of skeletal muscle tissues after exposure to conditioned media from the liver model.** (A) Schematic representation of 3D skeletal muscle fabrication, maturation and experimental timeline. CM, conditioned media; D, day. (B) Electrical pulse stimulation (EPS) regime and contractile profile. MASH, metabolic dysfunction-associated steatohepatitis; SL, steatotic liver. (C) Twitch-type contractions at 2 Hz. (D) Specific contractile forces at 2 Hz relative to the control mean. Differences between means tested by one-way ANOVA. (E) Tetanic-type contractions at 50 Hz. (F) Specific contractile forces at 50 Hz relative to the control mean. Differences between means tested by Kruskal–Wallis test. (G) Representative confocal images of 3D skeletal muscle tissue cryosections stained against sarcomeric α-actinin (SAA), F-actin and nuclei. White arrows point at representative myotube sizes. Scale bars: 50 μm; 10 μm (insets). (H) Diameter distribution of myotubes observed by confocal microscopy. Biological replicates: *n*=6 per experimental group. (I,J) Median myotube diameter (I) and area (J). Results are expressed as mean±s.e.m. Differences between means were tested by one-way ANOVA followed by Dunnett's post-hoc tests when appropriate. *P<0.05; **P<0.01.

By subjecting skeletal muscles to EPS, we functionally evaluated skeletal muscles in response to MASLD exposure, recapitulating contractions at increasing stimulation frequencies. Throughout the EPS protocol, skeletal muscles exerted progressively lower forces as liver disease severity increased, with MASH-CM having the strongest negative effect (Fig. 4B). At 2 Hz stimulation (twitch), SL-treated tissues contracted 29% weaker, and MASH-treated muscles showed a 60% decrease in contractility compared to that in controls, although differences did not reach statistical significance (*P*=0.06) (Fig. 4D). At 50 Hz stimulation (tetanic), the functional impairment became more pronounced: SL-treated tissues exerted 53.1% less force, while MASH-treated muscles displayed severe contractile deficit, with a 77.2% reduction in contractility compared to that of controls (*P*=0.01) (Fig. 4F). CM from LPS-alone-treated livers produced intermediate effects, with twitch and tetanic forces comparable to those in SL-treated tissues, although not significantly different from those of controls.

Histological evaluation of transversal cryosections revealed changes in myotube morphometry in both SL- and MASH-treated groups (Fig. 4G), with an overall leftward shift in size distribution (Fig. 4H). Quantification of myotube morphometry showed significantly reduced diameter (−6.5%, *P*=0.003) and cross-sectional area (CSA; −10.0%, *P*=0.02) in SL-treated myotubes compared to control myotubes (Fig. 4I,J). Surprisingly, MASH-CM did not cause significant reductions in diameter (−2.8%, *P*=0.22) and area (−6.0%, *P*=0.2), despite producing the most severe functional impairment. This indicates the involvement of mechanisms beyond fiber atrophy.

### Severe liver disease media alters muscle regeneration independently of proteolytic pathways

Given the significantly reduced myotube size in SL-treated tissues, we anticipated upregulation of classical atrophy markers F-box protein 32/atrogin-1 (*FBXO32*) and tripartite motif-containing 63 (*TRIM63*)/muscle RING finger protein 1 (MuRF1). Surprisingly, both *FBXO32* and *TRIM63* were significantly downregulated following exposure to diseased liver CM. SL-CM reduced *FBXO32* expression by 29.6% (*P*=0.02), while MASH-CM caused more pronounced suppression of both *FBXO32* (−57.6%, *P*<0.001) and *TRIM63* (−46.8%, *P*=0.02) (Fig. 5E,F). This pattern suggests that MASLD-induced muscle dysfunction operates through mechanisms distinct from canonical ubiquitin–proteasome-mediated atrophy.

**Fig. 5.**
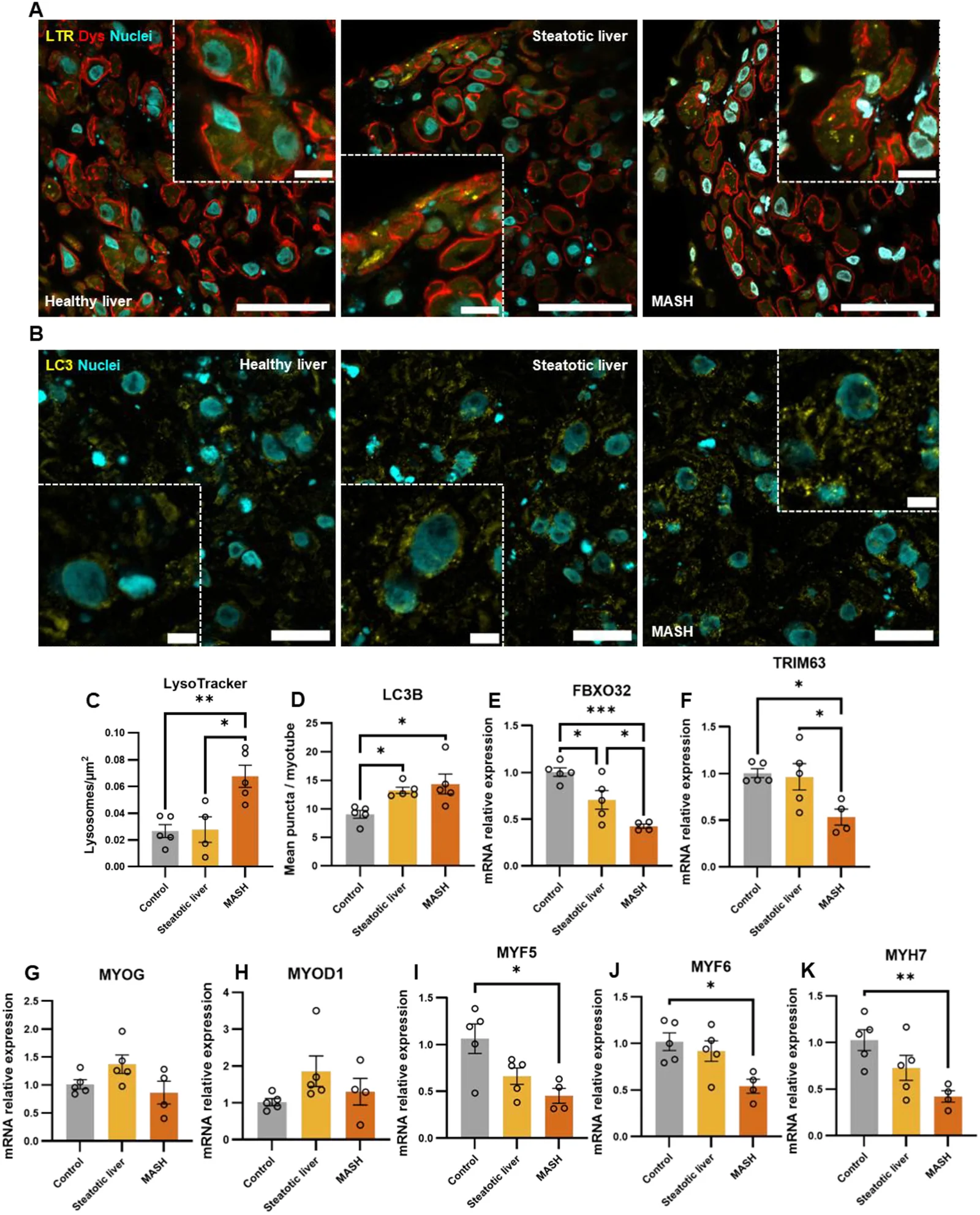
**Visualization of acidic compartments and LC3 puncta, and quantification of relative expression of 3D skeletal muscle tissues after exposure to conditioned media from the liver model.** (A) Representative confocal images of cryosections stained with an antibody against dystrophin, LysoTracker™ Red DND-99 and DAPI. Scale bars: 50 μm; 10 μm (insets). (B) Representative confocal images of 3D skeletal muscle tissue cryosections stained against LC3B protein. Scale bars: 20 μm; 50 μm (insets). (C) Quantification of LysoTracker staining. (D) LC3B^+^ puncta per myotube. (E-K) Relative expression of atrogenes *FBXO32* (E) and *TRIM63* (F), and myogenic regulatory factors *MYOG* (G), *MYOD1* (H), *MYF5* (I), *MYF6* (J) and *MYH7* (K) at the final timepoint, using the mean of *RPLP0* and *B2M* as the housekeeping genes. Expression levels were calculated by the ΔΔCt method. Results are expressed as mean±s.e.m. and were compared using one-way ANOVA followed by Tukey's or Dunnett's post hoc tests when appropriate. *P<0.05; **P<0.01; ***P<0.001.

Analysis of myogenic regulatory factors revealed a differential response to liver disease severity. Under SL conditions, myogenin (*MYOG*) and myogenic differentiation 1 (*MYOD1*) showed non-significant upward shifts (+35.5%, *P*=0.23 and +82.5%, *P*=0.17, respectively) (Fig. 5G,H), potentially reflecting compensatory regenerative signaling. However, MASH-CM failed to induce this response. Further examination of the myogenic factor family revealed significant suppression with MASH-CM treatment: myogenic factor 5 (*MYF5*) (−57.4%, *P*=0.01) and myogenic factor 6 (*MYF6*) (−46.9%, *P*=0.01) were both downregulated in inverse proportion to liver disease severity (Fig. 5I,J). Similarly, β-myosin heavy chain (*MYH7*) showed progressive downregulation across conditions, reaching significance with MASH treatment (−58.9%, *P*=0.008) (Fig. 5K). Together, these findings indicate that MASH-CM exposure alters the myogenic- and differentiation-associated transcriptional profile of mature muscle constructs, suggesting a stress-associated remodeling of myogenic regulatory networks that contributes to reduced muscle performance.

### MASLD exposure promotes excessive autophagy flux in skeletal muscle

To investigate whether autophagy contributes to the observed muscle dysfunction, we assessed autophagic flux using complementary approaches. LysoTracker™ Red DND-99 (LTR99), an acidotropic dye that labels lysosomes and autophagolysosomes, revealed distinct differences in autophagy activation across treatment groups (Fig. 5A). Control muscles showed a low, diffuse LTR99 signal evenly distributed throughout the sarcoplasm, whereas SL- and MASH-treated tissues displayed prominent punctate patterns indicative of autophagolysosome activation and accumulation. Quantification confirmed progressive autophagy activation: whereas SL-CM treatment increased puncta density non-significantly, MASH-CM induced a marked 153.5% increase (*P*=0.005), with significant differences between SL-CM and MASH-CM groups (*P*=0.01).

Autophagosome formation was assessed by LC3B (MAP1LC3B) immunostaining, detecting the lipidated form (LC3-II) that associates with autophagosomal membranes. LC3B puncta were visibly elevated in both intensity and number per myotube in diseased liver-treated tissues (Fig. 5B). Quantification revealed that SL-CM treatment increased LC3B puncta by 46.8% (*P*=0.03), while MASH-CM treatment produced a 59.0% increase (*P*=0.01), corroborating the LysoTracker findings. Together, these results demonstrate that factors secreted by diseased livers progressively activate autophagy flux in skeletal muscle, with activation intensity correlating with liver disease severity.

## DISCUSSION

MASLD is a continuum characterized by triglyceride accumulation alongside cardiometabolic risk factors. Progression to MASH signifies a transition toward lipotoxicity, inflammation and fibrogenesis (Tacke et al., 2024; Devasia et al., 2025). Capturing this transition *in vitro* is challenging, as it requires models that accurately represent both the multicellular interactions and architectural features of the hepatic niche, aspects that conventional screening platforms address partially (Boeckmans et al., 2018; Polidoro et al., 2021; Gatzios et al., 2022).

To meet these criteria while ensuring experimental robustness, we employed a human HepaRG/LX-2 co-culture embedded in a collagen-based hydrogel. HepaRG cells offer long-term metabolic stability and facilitate dual hepatocyte-biliary differentiation, thereby mitigating donor-to-donor variability associated with primary cultures (Andersson et al, 2012). Incorporating LX-2 stellate cells enabled us to model reciprocal paracrine signaling, which is a key factor of hepatocellular injury and fibrotic activation in MASH (Zahmatkesh et al., 2022). Compared to complex microfluidic systems, our hydrogel scaffold strikes a practical balance between physiological relevance and scalability. This design allows for multiplexed readouts without sacrificing throughput.

We chose NEFAs and LPS as a defined induction strategy rather than multifactorial metabolic syndrome–like cocktails. Although broader formulations more closely emulate concurrent metabolic dysfunction, defined stimuli enhance standardization and still align with physiological sources of hepatic lipid load, as peripheral lipolysis and dietary fatty acids represent the main contributors to hepatic lipid influx in MASLD (Lambert et al., 2014). In this context, LPS functions as a synergistic second hit reflecting gut–liver axis impairment and reliably accelerates the shift toward inflammation (Schilcher et al., 2023), while avoiding the batch variability introduced by complex mixtures (Bronsard et al., 2024).

Within this framework, our constructs mimicked multiple hallmarks of MASLD progression. Steatosis intensified across lipid readouts (Fig. 1C-E), with the most pronounced increase in the group co-treated with NEFAs+LPS, supporting a strong synergy between the two inducers. Because LPS alone did not induce steatosis (Fig. 1D,E), this effect appears to stem from treatment synergy rather than from HSC stimulation.

Additionally, *ALB* mRNA declined proportionally to injury severity (Fig. 1F), consistent with the loss of hepatocyte identity under metabolic stress. Human data demonstrate reduced albumin synthesis with worsening liver function, inversely proportional to Child–Pugh and model for end-stage liver disease (MELD) scores (Amouzandeh et al., 2023). Hepatocyte nuclear factor 4 alpha (*HNF4A*) positively controls and correlates with *ALB* expression (Guzman-Lepe et al., 2018) and is inhibited under lipotoxicity (Takagi et al., 2010), suggesting that the *ALB* downregulation is a plausible consequence of lipotoxicity through dedifferentiation in our constructs. Finally, AST, a canonical marker of hepatocellular damage, was elevated in the co-treated group (Fig. 1G), confirming that the model recapitulates hepatocellular injury (Ampuero et al., 2024). Control constructs showed noticeable baseline variability in albumin mRNA expression and AST release, consistent with the intrinsic heterogeneity of 3D multicellular hydrogel cultures; nevertheless, the NEFAs+LPS condition produced significant and biologically coherent changes in both readouts, supporting their use as complementary indicators of MASH-like hepatocellular dysfunction in this model.

A robust MASLD platform must capture HSC activation, a key event in the transition from steatosis to fibrotic injury. During this shift, quiescent vitamin-A-storing HSCs become proliferative and chemotactic myofibroblasts in response to various paracrine signals (Tsuchida and Friedman, 2017). Vimentin, an indicator of HSC activation, was observed to increase in length and intensity, particularly in the NEFAs+LPS condition, reflecting the histological changes seen in human MASLD (Fig. S2B). Although formal demonstration of HSC quiescence requires dedicated cell-cycle and quiescence marker validation, our data support enhanced HSC activation and fibrogenic remodeling relative to the baseline 3D co-culture state.

As HSCs transdifferentiate into myofibroblasts, they secrete chemokines and pro-inflammatory mediators that exacerbate injury. Consistent with the sequence of MASLD progression, NEFA exposure on day 4 did not change IL-6, IL-8 or PDGF-BB levels (Fig. 2B-D), indicating that lipid overload alone does not trigger early paracrine activation. However, once HSC activation occurred, these cytokines increased significantly, particularly IL-6 in the co-treated group (Fig. 2H-J), highlighting the role of stellate cells in hepatic interactions.

The elevated inflammatory and fibrogenic markers in our model indicate key mechanisms in MASLD progression. IL-6, crucial for hepatocyte health (Schmidt-Arras and Rose-John, 2016), was persistently high in the MASH-like group, reflecting chronic inflammation and correlating with clinical disease severity (Aleksandrova et al., 2014) (Fig. 2H). IL-8, linked to lipotoxicity and immune recruitment (Joshi-Barve et al., 2007), was significantly upregulated. It is a more specific indicator of cirrhosis and fibrotic injury than traditional markers (Liu et al., 2022). Its role in promoting angiogenesis suggests a connection to hepatocarcinogenesis and supports fibrosis progression (Kubo et al., 2024) (Fig. 2I). Additionally, PDGF-BB, a key driver of HSC activation (Borkham-Kamphorst and Weiskirchen, 2016), was notably elevated in the MASH-like group, highlighting the importance of PDGF signaling in fibrosis (Wada et al., 2024) (Fig. 2J). Because both HepaRG – owing to their cholangiocyte-like features – and HSCs express additional PDGF isoforms, our model allows exploration of PDGF-dependent pathways, enhancing understanding of pro-fibrotic signaling in MASLD.

Unlike cytokines, caspase-3 levels were already significantly elevated on day 4 (Fig. 2A), likely reflecting early hepatocyte apoptosis in response to lipotoxic stress. This increase persisted through day 7, consistent with the temporal profile of caspase-3 as both a marker and mediator of hepatocyte injury in MASH (Dong et al., 2021). Early emergence of apoptotic cues aligns with the established sequence in which lipotoxicity induces hepatocyte apoptosis, apoptotic bodies and damage-associated molecular patterns activate HSCs, and the inflammatory response is subsequently amplified, establishing a feed-forward injury loop.

Collagen fibers extending between adjacent cell clusters are a hallmark of bridging fibrosis *in vivo* (Hall et al., 2021), and we observed comparable long, aligned fibers in polarized images of construct cryosections (Fig. 3A,B). Together with increased Picrosirius Red staining and *COL1A1* upregulation, these findings suggest that the model reproduces key architectural features of fibrotic remodeling (Fig. 3G). Unexpectedly, PIIINP and, to a lesser extent, PINP levels were lower on day 7. This pattern may reflect distinct temporal dynamics of collagen species (Fig. 3C-F). Fibrosis was assessed using a targeted set of biomarkers rather than an unbiased discovery approach. Although collagen deposition, polarized-light birefringence, COL1A1 expression and collagen propeptide measurements show evidence of active ECM remodeling, broader tissue proteomics or fibrosis-focused transcriptomic panels would be required to resolve the full spectrum of fibrotic mediators generated by the liver constructs. Soluble PINP and PIIINP may reflect not only collagen synthesis but also matrix retention, local ECM assembly, diffusion and release dynamics through the hydrogel. Therefore, these markers were used as complementary indicators of collagen turnover rather than independent evidence of fibrosis. Further studies incorporating deeper histological, proteomic or transcriptomic characterization are needed to resolve matrix deposition and organization in greater detail. The variability observed in PINP and COL1A1 responses under treatment with NEFAs+LPS likely reflects heterogeneity in multicellular 3D matrix remodeling. Therefore, these fibrosis-associated readouts should be interpreted as targeted evidence of MASH-like remodeling rather than comprehensive profiling.

Together, we demonstrate that HepaRG/LX-2 hydrogels reproduce key molecular, inflammatory and architectural features of MASLD–MASH, while generating physiologically relevant secreted factors capable of communicating and influencing extra-hepatic tissues. These disease-stage-specific signals enable examination of how MASLD-derived factors affect skeletal muscle function and contribute to muscle-wasting.

Consistent with clinical evidence linking MASLD severity to impaired muscle function (Kumbaroğlu et al., 2025), mature 3D skeletal muscle tissues exposed to CM from SL- and MASH-like liver constructs exhibited reduced contractile capacity (Fig. 4B). This decline followed the severity-dependent pattern: SL-CM induced a marked reduction in contractility relative to controls, while MASH-CM further impaired contractility, particularly at higher stimulation frequencies. This frequency-dependent deficit is physiologically relevant, as it mirrors the exercise intolerance and premature fatigue reported in patients with MASLD (Kumbaroğlu et al., 2025). Notably, the functional decline between SL-treated and MASH-treated muscle tissues was more modest than the larger drop observed between controls and SL-treated muscle tissues, a non-linear pattern also described clinically and likely reflecting reduced sensitivity of muscle to escalating hepatotoxic cues at advanced disease stages (Fig. 4C-F).

Among the elevated mediators in our system, IL-6 trans-signaling appears particularly relevant. Although transient IL-6 supports regeneration and adaptation in muscle, systemic chronic elevation disrupts myogenic differentiation and promotes catabolism via JAK/STAT3 pathways (Visser et al., 2002; Tandon et al., 2021), leading to upregulation of muscle-specific E3 ubiquitin ligases *FBXO32*/atrogin-1 and *TRIM63*/MuRF1 (Huang et al., 2020). Sustained IL-6 can also interfere with IGF1 signaling, impairing muscle growth and protein degradation (De Benedetti et al., 1997). Beyond protein turnover, elevated IL-6 disrupts skeletal muscle redox balance by altering nuclear factor erythroid 2-related factor 2 (*Nrf2*)-dependent antioxidant defenses and activating autophagy–ubiquitin–proteasome pathways (Forcina et al., 2019). Consistently, we observed increases in lysosomal biogenesis and autophagic bodies in MASH-treated tissues.

Our data reproduce the clinical dissociation between muscle mass and functional decline (Henrot et al., 2023). SL-CM caused the greatest reduction in myotube size, demonstrating that MASLD-derived factors can directly induce wasting. In contrast, MASH-CM produced the most severe functional impairment despite the MASH-treated group exhibiting larger myotubes than those of the SL-treated group (Fig. 4D,F,I,J). This indicates that performance deficits arise not solely from reduced size but also from metabolic or molecular dysfunction, consistent with the clinical concept that muscle quality is a stronger determinant of outcomes than muscle mass alone.

Despite myotube size reduction, *FBXO32* and *TRIM63* were progressively downregulated with increasing MASLD severity (Fig. 5E,F). Although both genes are well-established mediators of proteasomal degradation during muscle atrophy (Bonaldo and Sandri, 2013), recent work demonstrates that their expression profiles vary substantially across atrophy models, and they are not upregulated in aging-associated sarcopenia (Hunt et al., 2021). Therefore, the observed *FBXO32* and *TRIM63* repression aligns with emerging evidence that metabolic disorders engage alternative MW pathways and suggests that MASLD-induced MW could follow distinct regulatory programs.

Elevated IL-8 in MASH-CM may contribute to this atypical response. Although pro-inflammatory and chemoattractant in liver, IL-8 exerts anti-catabolic effects by enhancing Akt phosphorylation, inactivating *Foxo3* and reducing expression of atrogin-1 and MuRF1 (Milewska et al., 2019). This may explain the paradoxical preservation of myotube size in MASH-treated muscles despite severe functional impairment, suggesting that IL-8 contributes to a compensatory strategy delaying atrophy during early hepatic stress. However, we speculate that prolonged exposure (>48 h) may eventually overwhelm this compensation.

Only muscles exposed to MASH-CM exhibited a significant increase in lysosome density, suggesting enhanced lysosomal biogenesis (Fig. 5A). Consistently, LysoTracker™ staining revealed alterations in acidic compartment size and organization, increasingly recognized as features of metabolic dysfunction (de Araujo et al., 2020) (Fig. 5A). Increased LC3B puncta following SL-CM and MASH-CM exposure may indicate enhanced autophagosome formation (Fig. 5B-D). Metabolic cues such as elevated free fatty acids disrupt autophagosome–lysosome cooperation (de Araujo et al., 2020; Gros and Muller, 2023), consistent with impaired terminal autophagic degradation and compensatory lysosomal biogenesis. However, LC3B accumulation alone cannot distinguish between increased autophagosome formation and impaired autophagic degradation; therefore, dedicated flux assays, such as lysosomal inhibition with chloroquine or bafilomycin A1, together with analyses of mitochondrial integrity, oxidative stress and mitophagy, will be required to deepen the interpretation of autophagy dysregulation (Tanida et al., 2008).

In SL-CM-exposed tissues, mild non-significant increases in *MYOG* and *MYOD1* suggest early compensatory repair responses, similar to the dysregulation reported in sarcopenia (Zammit, 2017; Brzeszczyńska et al., 2018; Pelosi et al., 2021; Park and Choi, 2023) (Fig. 5G,H). Modest IL-6 increases can promote myoblast commitment and *MYOD1*/*MYOG* transcription, while slight *MYF5* decreases suggest transition to *MYOD1*-driven differentiation. However, under MASH conditions, *MYOG* and *MYOD1* returned toward control levels, whereas *MYF5* declined further, consistent with repression of early myogenic programs in chronic inflammation (Brzeszczyńska et al., 2018) (Fig. 5I). *MYH7* was significantly reduced in MASH-treated muscles (Fig. 5K), indicating impaired terminal differentiation, similar to effects of pro-inflammatory CM from obese individuals (O'Leary et al., 2018) and LPS-treated murine cells (Ono and Sakamoto, 2017). Elevated PDGF-BB in MASH-CM likely contributes, as it actively inhibits myogenic differentiation while promoting proliferation (Yablonka-Reuveni et al., 1990; Lawlor et al., 2000). Together, these findings suggest that SL-derived signals initially trigger compensatory myogenic programs, but exposure to MASH-like milieu suppresses differentiation, contributing to impaired muscle quality and contractility, despite preserved myotube size. Because MASLD/MASH-associated muscle impairment occurs in mature skeletal muscle *in vivo*, CM exposure was performed after differentiation to assess the response of already formed human muscle tissues to the hepatic milieu. In this context, changes in *MYOD1*, *MYOG*, *MYF5*, *MYF6* and *MYH7* expression indicate altered myogenic/differentiation-associated transcriptional regulation in mature constructs. However, because this model does not include a characterized satellite-cell regenerative compartment, these changes do not imply impaired regenerative capacity. Protein-level validation and future models incorporating satellite-cell activation, proliferation and differentiation markers will be needed to determine whether these transcriptional alterations translate into impaired regeneration.

Our platform establishes NEFA-driven steatosis as a stressed hepatic state, while LPS addition accelerates transition to MASH-like stages characterized by hepatocellular injury (reduced *ALB*, elevated AST), sustained apoptotic signaling (elevated caspase-3), stellate-cell activation (vimentin remodeling), fibrogenic remodeling (collagen deposition, increased *COL1A1*) and escalated inflammatory/growth factor secretion (IL-6, IL-8, PDGF-BB). These features position liver constructs as sources of disease stage-specific factors transmitting graded hepatic stress signatures to other tissues without direct contact.

This liver-derived environment elicits multilayered muscle responses, including loss of muscle quality beyond pure atrophy. CM induce severity-dependent contractile decline (greatest under MASH), altered proteostasis (LC3B accumulation in SL/MASH; increased lysosome density predominantly under MASH), altered myogenesis and differentiation (downregulated *MYF5*, *MYF6*, *MYH7*), consistent with impaired maturation potentially reinforced by IL-8 and PDGF-BB signaling. Notably, canonical atrogene pathways were not engaged (downregulated *FBXO32*/*TRIM63*), and functional decline exceeded structural changes. This supports a framework in which progressive MASLD-like dysfunction drives MW through inflammatory/growth factor stress signaling, dysregulated autophagy–lysosomal homeostasis and altered differentiation programs, providing a modular approach to map timing, thresholds and intervention points in organ crosstalk.

### Clinical implications

By combining a MASLD–MASH liver module with a functional skeletal muscle module, this platform enables interrogation of causal liver–muscle communication *in vitro*. It directly links disease-stage-specific hepatic secreted factors to quantifiable muscle outcomes – performance, morphology and protein balance – readouts difficult to integrate in patient studies and screening systems.

### Limitations and future directions

In the current setup, we acknowledge the baseline variability observed in some experimental groups as a characteristic of 3D bioengineered systems. However, the magnitude of the response to the ‘two-hit’ stimulus (like the 3-fold increase in AST release) demonstrates that the model's sensitivity is not compromised by this biological noise. Our static 3D hydrogel model with induction by NEFAs+LPS improves standardization and multiplexing but cannot fully capture genetic and dietary MASLD drivers. Although we focused on IL-6, IL-8 and PDGF-BB as representative markers of the MASH-like inflammatory and fibrogenic secretome, we recognize that the liver–muscle crosstalk likely involves a broader set of mediators. The present marker panel should be interpreted as a targeted validation of key MASH-associated secretory features rather than a comprehensive definition of the disease-conditioned secretome. The modularity of this human 3D platform is designed to facilitate future high-resolution proteomic, cytokine, metabolomic and lipidomic profiling of the CM to uncover novel drivers of MW. CM dilution (1:50) minimized confounding effects of liver culture medium; future designs should enable tissue-specific media optimization while preserving paracrine signaling. Although reduced contractile force and morphometric changes support a muscle-quality-dominant phenotype consistent with clinical observations in sarcopenia and MASLD-associated muscle impairment, future studies incorporating mitochondrial integrity, oxidative stress, oxygen-consumption analysis, ATP production and metabolic profiling will be required to determine how bioenergetic alterations contribute to the observed functional decline. Regarding protein homeostasis, LC3B puncta accumulation cannot distinguish autophagy initiation from impaired flux, requiring flux assays and broader profiling of mitochondrial and lysosomal function. Future work must extend crosstalk duration, incorporate patient-derived cells, add immune and adipose components, and advance muscle phenotyping while preserving modularity to systematically test interventions at meaningful throughput.

### Conclusions

Using a human HepaRG/LX-2 hydrogel co-culture and induction with NEFAs+LPS, we established a MASLD–MASH phenotype in three dimensions. The liver module produces factors dependent on disease stage, offering a controlled, human-derived and scalable source of hepatic cues for investigating extra-hepatic complications. CM from diseased liver constructs were sufficient to impair mature 3D skeletal muscle *in vitro*, inducing severity-dependent loss of contractile performance with a marked frequency-sensitive deficit, alongside changes consistent with altered proteostasis and myogenic regulation, and structural changes. Notably, functional decline exceeded changes in myotube size and occurred without canonical atrogene induction, emphasizing a muscle-quality-dominant phenotype and highlighting the value of integrating functional outputs into liver–muscle axis models. Finally, the platform's modular design provides a clear path to incorporate additional drivers of the axis – immune and adipose components, longer exposure paradigms and muscle-specific features such as myosteatosis – while preserving the throughput needed for systematic perturbation and intervention testing.

## MATERIALS AND METHODS

### Cell culture

#### Immortalized hepatic cells

HepaRG™ cells (HPRGC10, Thermo Fisher Scientific, Waltham, MA, USA) were cultured in hepatocyte growth medium (HGM) consisting of William's E Medium+GlutaMAX™ supplement (Gibco, Grand Island, NY, USA), 10% fetal bovine serum (FBS; 10270-106, Gibco), 1% penicillin–streptomycin (10,000 U ml^−1^; 15140-122, Thermo Fisher Scientific), 5 μg ml^−1^ human insulin (I2643, Sigma-Aldrich, St Louis, MO, USA) and 50 μM hydrocortisone 21-hemisuccinate sodium salt (H4881, Sigma-Aldrich) at 37°C in a humidified atmosphere with 5% CO_2_. Subculturing was carried out by trypsinization as instructed in the user manuals.

#### Hepatic stellate cells

LX-2 cells (SCC064, Merck, Darmstadt, Germany) were cultured as instructed by the supplier, in Dulbecco's modified Eagle medium (DMEM) with GlutaMAX™, 4.5 g/l D-glucose, without sodium pyruvate (61965-026, Gibco), supplemented with 1% penicillin–streptomycin (10,000 U ml^−1^; 15140-122, Thermo Fisher Scientific). Additional 10% and 2% FBS (10270-106, Thermo Fisher Scientific) were used for trypsin deactivation and routine growth, respectively.

#### Human myoblasts

Cell line AB1079 (male, aged 38) from quadriceps was kindly provided by Dr Eduard Gallardo at the Sant Pau Hospital, Barcelona, Spain. Informed consent was obtained from the cell donor, and clinical investigation was conducted according to the principles expressed in the Declaration of Helsinki. Cells were handled according to standard procedures, using skeletal muscle cell growth medium (SkM GM; C23060, Promocell, Heidelberg, Germany) supplemented with mix for skeletal muscle cells [SupplementMix (C23060, Promocell), 10% FBS (10270-106, Thermo Fisher Scientific) and 100 U ml^−1^ 1% penicillin–streptomycin (10,000 U ml^−1^; 15140-122, Thermo Fisher Scientific)]. Differentiation was carried out in DMEM with GlutaMAX™, 4.5 g/l D-glucose, without sodium pyruvate (61965-026, Gibco), with 1% KnockOut Serum Replacement (KO-SR; 10828028, Thermo Fisher Scientific), 1% penicillin–streptomycin–glutamine (PSG; 10378-016, Gibco) and insulin–transferrin–selenium–ethanolamine supplement (ITS-X; 51500-056, Gibco), referred to as SkM DM.

### Preparation of NEFAs

A palmitic acid (PA) stock was prepared by dissolving PA powder (P0500, Sigma-Aldrich) in 2-propanol (131090.1212, PanReac AppliChem, ITW Reagents, Darmstadt, Germany) to a 400 mM concentration. Oleic acid (OA; O1008, Sigma-Aldrich) was diluted in 2-propanol to a 400 mM concentration. Both NEFA solutions were aliquoted and stored at −20°C. PA and OA were mixed at a 2:1 volume ratio to obtain a concentrated PA/OA mixture. Because NEFAs require a carrier to enter the cell, the PA/OA mixture was diluted 1:100 in 1% bovine serum albumin (BSA; fatty acid-free; A8806, Sigma-Aldrich) in 1× PBS. BSA is a commonly used carrier for NEFA formulations. The resulting NEFA–BSA solution, hereafter referred to as the NEFA stock, had a NEFAs/BSA molar ratio of 4:1. Aliquots were stored at −20°C.

### High-lipid regime for HepaRG in two dimensions

HepaRG cells were seeded in tissue culture-treated 96-well plates at a density of 5.0×10^4^ cells cm^−2^ and maintained for 24 h in HGM at 37°C in a humidified 5% CO_2_ incubator. After 24 h, the medium was replaced with serum-free medium (SFM) composed of William's E medium (GlutaMAX™ supplement) supplemented with HepaRG™ Induction Medium Supplement (5×; HPRG740, Gibco). Cells were allowed to adapt in SFM for an additional 24 h before induction. Thereafter, cells were challenged with 400 μM palmitic acid/oleic acid (2:1) NEFA mixture, prepared as described above, diluted in SFM, and incubated for 24, 48 or 72 h before endpoint assays.

### Metabolic activity/viability and lysosomal activity assays

#### MTS assay

At 24, 48 and 72 h after lipid challenge, the culture medium was removed, and cells were washed three times with 1× PBS for 5 min. Wells were then filled with 100 μl fresh SFM supplemented with 20 μl CellTiter 96^®^ AQueous One Solution Cell Proliferation Assay reagent (G3580, Promega, Madison, WI, USA) and incubated at 37°C in a humidified atmosphere with 5% CO_2_ for 2 h. Absorbance at 490 nm was measured using a SPARK microplate reader (Tecan, Männedorf, Switzerland) after shaking for 5 s, 1 mm amplitude and 1440 rpm. Wells containing reagent but no cells were included as blanks to correct for background absorbance.

#### NR assay

A concentrated 1000× NR stock solution was prepared by dissolving Neutral Red powder (N4638, Sigma-Aldrich) at 4 mg ml^−1^ in 1× PBS. One day before each timepoint, a 1× NR working solution was prepared by diluting the stock in 1× PBS and incubating it overnight at 37°C to allow dye saturation. At 24, 48 and 72 h after lipid challenge, the working solution was centrifuged at 1000 ***g*** for 5 min and filtered through a 0.22 μm membrane to remove dye crystals. The culture medium was removed, and cells were washed three times with 1× PBS for 5 min. Wells were filled with warm, crystal-free NR working solution (1×) and incubated at 37°C in a humidified atmosphere with 5% CO_2_ for 2 h. The dye solution was then discarded, and wells were washed twice with 1× PBS. Finally, cells were incubated with 200 μl de-staining solution (50% v/v ethanol 96%, 49% v/v ultrapure H_2_O, 1% v/v glacial acetic acid). Absorbance at 540 nm was measured using a SPARK microplate reader (Tecan) after shaking for 5 s (1 mm amplitude, 1440 rpm). Wells containing reagent but no cells were included as blanks to correct for background absorbance.

### Fabrication of PDMS casting molds

Polydimethylsiloxane (PDMS) casting molds for skeletal muscle tissues were fabricated as described previously (Fernández-Costa et al., 2023). In brief, the mold design consists of a rectangular seeding chamber flanked by two flexible PDMS pillars. Following the established procedure, 3D printed master molds were used to generate negative molds, which were silanized with trichloro(1H,1H,2H,2H-perfluorooctyl)-silane (PFOTS). Final PDMS casting molds were prepared by casting PDMS prepolymer at a 10:1 ratio, curing at 80°C and thoroughly washing after demolding.

### Cell encapsulations

#### Fabrication of 3D liver constructs

We followed a 3D hydrogel coculture approach to model the human liver. Collagen type I-based hydrogels were fabricated with the components of PhotoCol with LAP kit (5170, Advanced BioMatrix, Carlsbad, CA, USA) to encapsulate cocultures of HepaRG™ and LX-2. This strategy combines thermal gelation with photoinitiator crosslinking, allowing easy modulation of the hydrogel's mechanical properties by adjusting collagen concentration, photo-crosslinking time and UV light energy. Both cell lines were trypsinized, counted following supplier instructions and mixed at a 2:1 ratio (HepaRG™:LX-2). Then, an encapsulation pre-mix was carefully prepared in ice, sequentially adding neutralizing solution (5205, Advanced BioMatrix), 1 M sodium hydroxide (NaOH; S2770, Sigma-Aldrich), 5 mg ml^−1^ reconstituted collagen solution (5198, Advanced BioMatrix) and 17 mg ml^−1^ lithium phenyl(2,4,6-trimethylbenzoyl)phosphonate (LAP; 5269, Advanced BioMatrix) solution in William's E Medium, protected from light. Next, HepaRG™/LX-2 cell suspension in HGM was added to the pre-mix and briefly homogenized. Final cell density in hydrogels was 15,000,000 cells ml^−1^. 30 μl drops of the homogenized encapsulation mix were then carefully pipetted on cell culture-treated 24-well plates, avoiding bubbles. Thermal gelation was achieved by incubating the drops at 37°C for 10 min, followed by a UV-light exposure for 30 s in a UVP Crosslinker (CL-1000 Analytik Jena, Jena, Germany), pre-heated for 30 s. Once a change in opacity and color was observed, all wells were filled with HGM, and the crosslinked 3D hydrogels were incubated at 37°C in a humidified atmosphere with 5% CO_2_.

#### Fabrication of 3D bioengineered skeletal muscle tissues

3D human skeletal muscle tissues were fabricated in PDMS casting molds as described elsewhere (Tejedera-Villafranca et al., 2023; Mughal et al., 2024). The casting molds, consisting of a rectangular-shaped pool and two vertical flexible PDMS pillars, were first cleaned by sonication in water with detergent, 2-propanol and Milli-Q water, followed by UV sterilization for 15 min. To minimize cell adhesion to the PDMS, molds were treated with 1% (w/v) Pluronic^®^ F-127 (Sigma-Aldrich) in cold phosphate-buffered saline (PBS; 100 μl per well) at 4°C for 2 h and rinsed with sterile PBS before use. Human myoblasts AB1079 were trypsinized, resuspended in SkM GM, and encapsulated at a density of 2.5×10^7^ cells ml^−1^ in a hydrogel mixture consisting of 30% (v/v) Matrigel^®^ Growth Factor Reduced Basement Membrane Matrix (356231, Corning, New York, NY, USA), 4 mg ml^−1^ fibrinogen from human plasma (F3879, Sigma-Aldrich) and 2 U ml^−1^ thrombin from human plasma (T6884, Sigma-Aldrich). Hydrogel casting was performed using pre-cooled material to prevent uncontrolled Matrigel polymerization. The mixture was carefully distributed evenly between the PDMS pillars and polymerized for 30 min at 37°C. Following polymerization, SkM GM supplemented with 1 mg ml^−1^ 6-aminocaproic acid (ACA; A2504, Sigma-Aldrich) was added to prevent fibrin degradation. Tissue compaction was visually monitored from this point forward to follow cell alignment and distribution of tension. After 48 h, cultures were switched to SkM DM containing 1 mg ml^−1^ ACA. Only half of the SkM DM volume was renewed every other day, and tissues were maintained under these conditions until the treatment stage.

### Recapitulation of steatosis and steatohepatitis in 3D livers

To mimic the progressive nature of MASLD, we formulated two treatment media. The first one included NEFAs to induce the steatotic liver phenotypes, while the second one contained NEFAs and LPS, a stimulator of HSCs, to address the progression to MASH. The medium for treatment with NEFAs was prepared by diluting the NEFA stock 1:100 in SFM. The medium for treatment with NEFAs+LPS was prepared by diluting the NEFA stock 1:100 in SFM and adding 0.1 μg ml^−1^ LPS (L8274, Sigma-Aldrich). As carrier control, BSA was diluted in SFM to match its molar concentration in the treatment media, and SFM alone was used for the untreated 3D livers as a control group. All 3D constructs were transitioned to SFM for 3 days to preserve the differentiated phenotypes and metabolic signatures of both cell types. Next, they were incubated in the respective treatment medium (control group, carrier control, NEFAs) for 4 days at 37°C in a humidified atmosphere with 5% CO_2_. On day 4, all treatment media were renewed. Only the formulation for the group modeling steatohepatitis was changed to NEFAs+LPS. All 3D livers were incubated under the same conditions for 3 additional days until the final timepoint.

### Treatment of skeletal muscle tissues with CM from 3D livers

At day 16 in differentiation conditions, half of the SkM DM volume was renewed, containing CM from 3D livers at 2× concentration, so that a 1× concentration was achieved after combining with the other half volume. Preliminary experiments show a toxic effect of liver cell culture media; therefore, several dilutions were tested. A final volume ratio of 1:50 liver components in SkM DM was determined to cause minimal effects on skeletal muscle tissues at a functional level. CM from the 3D livers were prepared as pooled mixes of equal volume of five liver biological replicates and used to treat all skeletal muscle biological replicates. To avoid media-composition bias, CM from all liver experimental groups, including control liver constructs, were diluted into SkM DM at the same final proportion. Thus, any dilution of SkM DM supplements, including ITS-X-derived insulin, transferrin and selenium, was equivalent across all muscle treatment groups. The final concentration of SkM DM and its supplements was therefore matched across control-CM, SL-CM and MASH-CM conditions.

### Tissue staining, immunohistochemistry and bioimage analysis

#### Oil Red O staining

For lipid accumulation analysis, cells were washed three times with 1× PBS for 5 min and fixed with 200 μl neutral buffered formalin solution, 10% (HT501128, Sigma-Aldrich) for 15 min under a fume hood. After two washes with ultrapure water (5 min each), cells were rinsed with 60% 2-propanol (v/v) and stained with freshly prepared Oil Red O solution (3:2 in ultrapure water; O1391, Sigma-Aldrich) for 15 min. Excess dye was removed by repeated washing with ultrapure water until the supernatant was clear. Nuclei were counterstained with Harris Modified Hematoxylin Solution (HHS16, Sigma-Aldrich) for 1 min, followed by extensive washing with ultrapure water to avoid precipitate formation. Samples were maintained in ultrapure water for imaging.

#### AdipoRed™ staining and quantitative analysis

To assess intracellular triglyceride accumulation in live 3D livers, AdipoRed™ Assay Reagent (PT-7009, Lonza Bioscience, Basel, Switzerland) was diluted to 3% (v/v) in 1× Live Cell Imaging Solution (A14291DJ, Invitrogen, Carlsbad, CA, USA) according to supplier instructions. Five 3D liver constructs per condition were washed three times with sterile 1× PBS, transferred to μ-Slides (80826, Ibidi, Gräfelfing, Germany) filled with diluted AdipoRed™ solution and 0.1% Hoechst 33342, trihydrochloride trihydrate, 10 mg/ml stock solution (H3570, Thermo Fisher Scientific). After 30 min incubation at 37°C in a humidified atmosphere with 5% CO_2_, constructs were removed from the incubator, and *z*-stacks (0.81 μm per slice) per construct were acquired using a confocal microscope LSM 800 (Carl Zeiss Microscopy GmbH, Jena, Germany) with a 20× objective (0.8 NA) and pinhole set to 1 Airy unit (AU), under appropriate incubation conditions. For analysis, processing and quantification, we used Fiji, a distribution of ImageJ (Schindelin et al., 2012). All z-stacks were converted to a sum projection with an equal slice number (15 slices). A custom ImageJ Macro was applied to segment the projected 2D images by Gaussian background subtraction, thresholding and mask generation. Mean gray values and areas were measured for segmented lipid droplets and nuclei. Area quantification results were normalized to nuclei number.

#### LIVE/DEAD staining and quantitative analysis

Cell-laden hydrogels were washed three times with 1× PBS under agitation. Next, the constructs were incubated in a dye solution containing 2 μM calcein AM, 4 μM ethidium homodimer-1 and 0.1% Hoechst prepared as per supplier instructions (LIVE/DEAD™ Viability/Cytotoxicity Kit, L3224, Invitrogen) for 25 min at 37°C in a humidified atmosphere with 5% CO_2_ and then imaged at the confocal microscope with a 10× objective (0.3 NA) and pinhole set to 1 AU, under appropriate incubation conditions. Image analysis was performed in Fiji, a distribution of ImageJ (Schindelin et al., 2012).

#### Cryosectioning

3D constructs were washed three times with sterile 1× PBS for 5 min, and fixed in 10% formalin solution (HT501128, Sigma-Aldrich) for 30 min at room temperature (RT). After fixation, tissues were washed three times in 1× PBS for 5 min and transferred to 30% (w/v) sucrose in 1× PBS at 4°C for 48 h. Constructs were then embedded in optimal cutting temperature (OCT) compound (P0091, Sigma-Aldrich) in plastic Cryomolds^®^ (VWR) and snap frozen by immersion in precooled isopentane previously chilled in liquid nitrogen. Blocks were stored at −20°C until further use. Tissue sections (20 μm) were obtained using a Leica CM1860 cryostat and collected on SuperFrost Plus™ Adhesion slides (11950657, Thermo Fisher Scientific). Sections were stored at −20°C until staining.

#### Standard immunofluorescence staining

Tissue cryosections were permeabilized with PBST (0.1% Triton X-100 in PBS) for 15 min at RT, washed three times with 1× PBS for 5 min, and incubated in blocking solution (sc-516214, Santa Cruz Biotechnology, Dallas, TX, USA) for 1 h at RT. Primary antibodies diluted in blocking buffer were applied overnight at 4°C. After washing three times with 1× PBS for 5 min, samples were incubated with fluorescently labeled secondary antibodies (Table S1) in blocking buffer, together with a cell structure probe, if necessary (Table S1) for 45-60 min at RT, protected from light. Following incubation, sections were washed three times with PBST for 5 min. Nuclear staining was performed with DAPI (Thermo Fisher Scientific) for 15 min at RT, protected from light, or with VECTASHIELD^®^ PLUS Antifade Mounting Medium with DAPI (H-2000, Vector Laboratories, Newark, CA, USA). After final washes in 1× PBS, slides were mounted with antifade mounting medium, coverslipped and sealed for imaging.

#### Picrosirius Red staining

To visualize collagen fibers inside the 3D livers, Picrosirius Red staining was performed as per the supplier's protocol. Briefly, liver tissue cryosections were hydrated in distilled water and then covered with Picrosirius Red solution for 1 h. Slides were quickly rinsed twice in 0.5% acetic acid solution, followed by one rinse in absolute ethanol. Two more changes of absolute ethanol were performed to completely dehydrate the samples. Organo/Limonene Mount™ (O8015, Sigma-Aldrich) was employed for mounting. Brightfield and polarized light microscopy were carried out at a Leica widefield DMRB upright microscope (Leica Microsystems GmbH, Wetzlar, Germany) equipped with a color camera.

#### LysoTracker™ staining

To visualize cellular acidic compartments, live skeletal muscle tissues were washed with 1× PBS and then incubated in 1 μM LysoTracker™ Red DND-99 (L7528, Invitrogen) probe solution for 30 min at 37°C. Then, tissues were washed three times with 1× PBS before fixation in 10% formalin solution (Sigma-Aldrich) for 30 min at RT. Three more washes with 1× PBS were carried out after fixation, and tissues were then stored at 4°C until further use. Cryosections pre-stained with LysoTracker™ were cryosectioned as described above and then stained with rabbit anti-dystrophin IgG antibody (ab15277, Abcam, Cambridge, UK; 1:200), followed by an incubation with donkey anti-rabbit IgG (H+L) AlexaFluor™ 647 (A31573, Invitrogen; 1:200). Nuclei were stained with VECTASHIELD^®^ PLUS Antifade Mounting Medium with DAPI (H-2000, Vector Laboratories). Slides were coverslipped and sealed for imaging. Sample imaging was performed at a confocal microscope LSM 800 (Carl Zeiss Microscopy GmbH) with a 40× oil objective (1.4 NA) and pinhole set to 1 AU. Acidic compartments were detected by sequential background subtraction, convolution filtering, thresholding and binary mask generation with a custom ImageJ Macro. Particles between 0.1 and 5.0 μm^2^ were analyzed. Segmentation accuracy was confirmed by overlaying masks with the original images. Total particle counts per image were normalized to myotube area, which was delineated using deep learning-based segmentation tool Cellpose 3 (Stringer et al., 2021) on dystrophin-stained channels. Feret diameters of myotubes were extracted with the LabelsToRois (Waisman et al., 2021) ImageJ plugin. Five biological replicates per condition were analyzed in technical triplicates.

#### LC3B immunostaining

We followed a protocol published elsewhere (Sabater-Arcis et al., 2021) with modifications. Briefly, skeletal muscle tissue cryosections were permeabilized with 100% methanol for 15 min at −20°C and incubated in blocking buffer [1× PBS, 0.5% BSA, 1% normal goat serum (S-1000, Vector Laboratories s)] for 1 h at RT. Samples were incubated overnight with rabbit anti-LC3B primary antibody (ab51520, Abcam; 1:200) in blocking buffer at 4°C. Next, cryosections were incubated with goat anti-rabbit IgG (H+L) AlexaFluor™ 568 (A-11036, Thermo Fisher Scientific; 1:200) for 2 h. Three washes with 1× PBS were performed between antibodies and nuclei counterstaining. All samples were mounted with VECTASHIELD^®^ PLUS Antifade Mounting Medium with DAPI (H-2000, Vector Laboratories), coverslipped and sealed for imaging.

### Albumin, AST, PINP and PIIINP measurements

To evaluate markers of MAFL and MASH, the frozen supernatants collected from the 3D livers were measured at the Hospital Clinic (Barcelona, Spain) facilities. A Cobas c 311/501 analyzer was used to measure albumin (CPS_000014) and AST (CPS_000040), as published elsewhere (De Chiara et al., 2022). PINP and PIIINP were quantified in an automated immunoassay analyzer (IDS-iSYS) with cartridges based on enzymatic assays.

### Cytokine measurements

Cell-culture supernatants were collected, centrifuged at 1000 ***g*** for 5 min to remove debris, aliquoted and stored at −80°C. On the day of assay, samples were thawed on ice. Cytokines were quantified using custom ProcartaPlex magnetic bead panels (Thermo Fisher Scientific) – 3-plex (human IL-6, IL-1β, TNF-α; PPX-03-MXKA6FD) and 4-plex (human caspase-3, CRP, IL-8, PDGF-BB; PPX-04-MXMFZDP), designed for 96-well plate setting – following supplier instructions. Briefly, standards were reconstituted and pooled with SFM as diluent, and then prepared by serial dilutions, with SFM as a blank sample to generate an eight-point curve. 50 μl mixed magnetic capture beads were added per well, washed using a hand-held magnetic plate washer, and incubated with 50 μl standards, controls or samples (run in duplicate) for 2 h at RT on a plate shaker (600 rpm), protected from light. Wells were washed two times with 1× wash buffer, and 25 μl biotinylated detection antibody mix was added and incubated for 30 min at RT with shaking. After washing twice, 50 μl streptavidin-PE was added and incubated for 30 min at RT with shaking, protected from light. Beads were rewashed twice, resuspended in 120 μl 1× reading buffer and acquired on a Luminex MAGPIX instrument. The beads/analyte counted per well were recorded, and median fluorescence intensity (MFI) values were fitted to five-parameter weighted logistic (5-PL) standard curves using xPONENT and ProcartaPlex Analysis App (Thermo Fisher Scientific) software. Analyte concentrations were verified to fall within each analyte's dynamic range.

### Electrical pulse stimulation, video analysis and functional assessment

Differentiated 3D skeletal muscle tissues were subjected to EPS after the treatment stage to induce contractile activity. A custom-made 24-well plate stimulation platform (Ruiz-Gutiérrez et al., 2025) with two graphite electrodes per well was connected to a pulse generator (Multifunction Generator WF1974, NF Corporation) and placed inside the XL S1 incubation chamber of a Zeiss Axio Observer Z1/7 microscope (37°C, 5% CO_2_). Tissues were maintained in fresh differentiation medium/ACA during stimulation. To induce twitch and tetanic contractions, EPS was delivered as square-wave pulses for 10 s at 1 V mm^−1^ field strength, 1 ms pulse width, and frequencies ranging from 1 to 50 Hz with a 10% duty, while 140 s long time series (1 ms exposure time, 82.3 frames s^−1^) focusing the top edge of the pillars were acquired using a 10× objective. Brightfield images of whole tissues (2.5× objective) were acquired under the same setup.

Fiji (Schindelin et al., 2012) was used to measure the displacements by finding the edges of the pillar and monitoring their intersection along a hand-drawn straight line perpendicular to the edge of the pillar. Next, Euler–Bernoulli's beam bending equation was employed to find the forces exerted to achieve the observed displacements as follows:

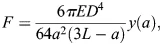
where ‘*F*’ is the exerted force, ‘*E*’ is the Young's modulus of the PDMS (1.6 MPa), ‘*D*’ is the diameter of the pillar, ‘*a*’ is the tissue height on the pillar, ‘*L*’ is the height of the focused point on the pillar, and ‘*y*(*a*)’ is the pillar displacement. The forces exerted by the tissues on the pillars were normalized to the cross-sectional area (CSA) of each tissue, resulting in specific forces per sample, as in

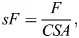
where ‘*sF*’ is the specific force.

### Real-time reverse transcription quantitative PCR

Whole 3D liver and skeletal muscle tissues were homogenized in 1 ml QIAzol reagent using a TissueLyser (50 Hz, 8 min) with stainless steel beads under RNase-free conditions, and RNA was purified using an miRNeasy Micro Kit (Qiagen) following the manufacturer's instructions. RNA concentration and purity were verified by Nanodrop (Thermo Fisher Scientific), and all samples complied with accepted quality criteria. Samples were stored at −80°C for long-term storage. Genomic DNA digestion and reverse transcription were performed using the QuantiTect Reverse Transcription Kit (Qiagen, 205311) with 1 μg total RNA as input. Reverse transcription quantitative PCR was performed using TaqMan probes (Table S2, listed in the Supplementary Materials and Methods) and TaqMan^®^ Fast Advanced Master Mix (4444557, Thermo Fisher Scientific). For liver tissues, *ACTB* was used as an endogenous control, while for skeletal muscle tissues, the geometric mean of *RPLP0* and *B2M* was used, after verifying their stability across samples and treatment groups. Reactions were run in technical triplicates with five biological replicates per experimental group, with no-amplification controls included per probe. Relative expression levels were calculated using the ΔΔCt method, normalized to the corresponding reference genes and control group. Follow-up multiple comparisons were calculated to assess differential expression with corrections if applied.

### Statistical analysis

All statistical analyses and graphs were performed in GraphPad Prism 10 (GraphPad Software, San Diego, CA, USA). Data distribution was assessed using the Shapiro–Wilk normality test. Outliers were identified using the ROUT method (Q=1%) and excluded when justified. For normally distributed data, comparisons among more than two groups were performed by one-way ANOVA, followed by post hoc multiple-comparisons tests (Tukey's test for all-pairwise comparisons or Dunnett's test when comparing groups against a single control). For datasets not following assumptions of parametric tests, an appropriate transformation was performed, or non-parametric alternatives were applied instead (Kruskal–Wallis with Dunn's multiple-comparisons) when criteria were not met after transformation. Data are presented as mean±s.e.m. on plots unless stated otherwise, and the number of independent biological replicates is reported in each figure legend (technical replicates are indicated per assay). Statistical significance was considered at *P*<0.05; exact *P*-values and details of each test are reported in the figure legends or Fig. S2.

## Supplementary Material

10.1242/dmm.052820_sup1 Supplementary information
