# Supplementary material for: A bioengineered platform reveals progressive impairment of skeletal muscles by metabolic dysfunction-associated steatotic liver disease-derived factors
Source: Dis Model Mech. 2026 Sep 17;19(9):dmm052820. doi: 10.1242/dmm.052820 (PMC13617984; doi:10.1242/dmm.052820)
Supplement: Supplementary file 1 [file dmm-19-052820-s1.pdf]

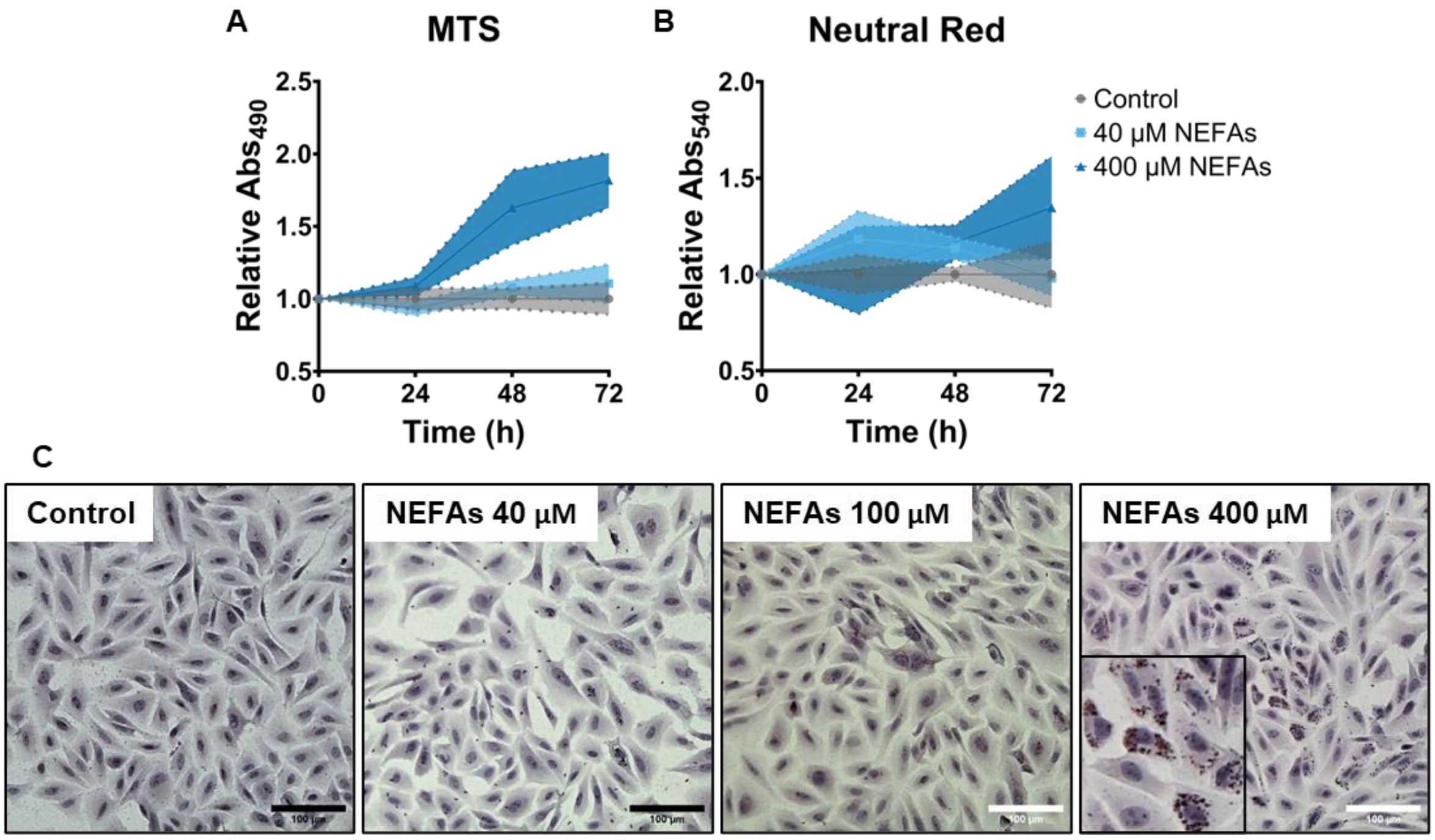

Fig. S1. Validation of lipid internalization by HepaRG cells in 2D culture. A) Metabolic activity measured by MTS assay at 24, 48, and 72 h following exposure to non-esterified fatty acids (NEFAs) (*n*=3). B) Lysosomal activity assessed by Neutral Red assay at 24, 48, and 72 h after NEFAs treatment (*n*=3). C) Representative brightfield images showing intracellular lipid droplets in HepaRG cells stained with Oil Red O and counterstained with Harris-modified hematoxylin. Scale bar = 100 µm.

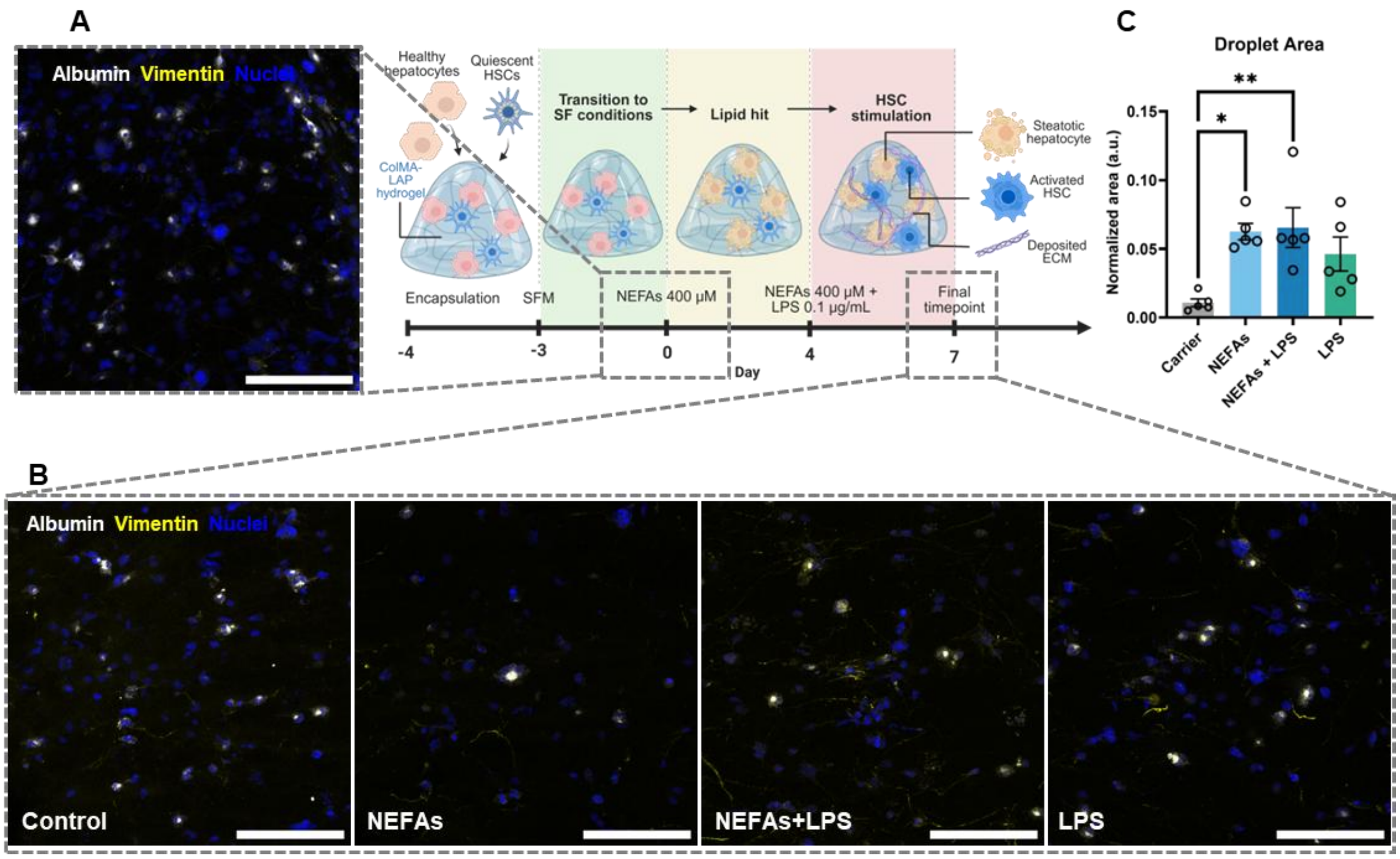

Fig. S2. Representative confocal Z-projections of construct cryosections stained with antibodies against hepatocyte marker albumin, intermediate filament protein vimentin, and DAPI (nuclei). A) Construct cryosections after the transition to serum-free conditions. B) Construct cryosections at the final timepoint. Scale bar = 100 μm. C) Lipid droplet area at the final timepoint normalized to nuclei number. Results are expressed as mean ± SEM and compared using one-way analysis of variance (ANOVA) followed by Tukey's post hoc tests when appropriate. * $p < 0.05$; ** $p < 0.01$.

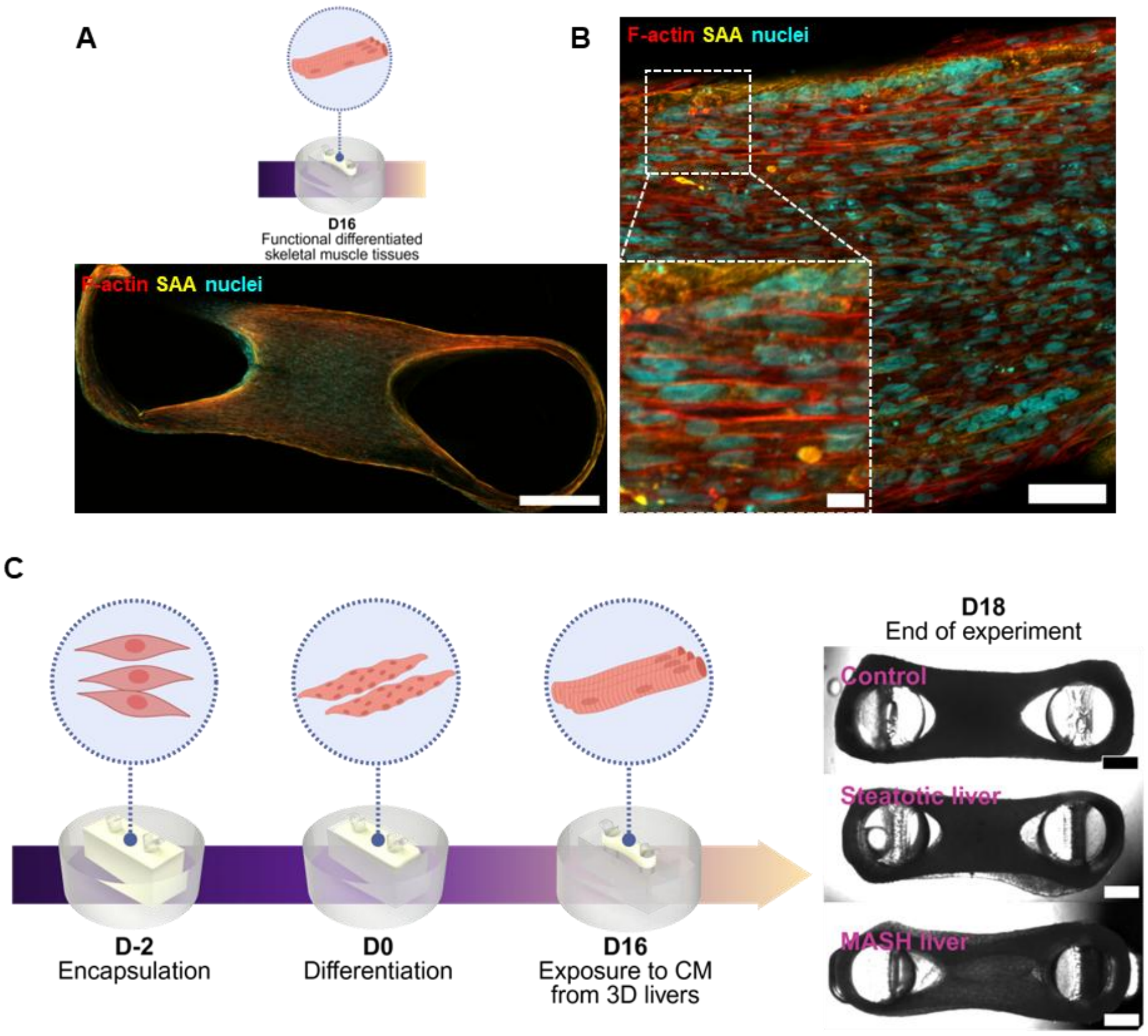

Fig. S3. Inspection of whole mounts of 3D skeletal muscles. A) 3D tissues at the end of the maturation stage. Scale bar = 500 µm. B) Representative confocal image of myotubes in a longitudinal view. Scale bar = 100 µm. Inset scale bar = 20 µm. C) Representative brightfield images of 3D tissues after exposure to conditioned media from the liver model. Scale bar = 500 µm.

Table S1. Antibodies and cellular probes.

Antibody or probe	Target	Supplier data	Dilution
**Rabbit anti-Dystrophin IgG antibody**	Dystrophin	ab15277, Abcam	1:200
**Anti-LC3B antibody from rabbit**	LC3B	ab51520, Abcam	
**Albumin Polyclonal Antibody from rabbit**	Albumin (ALB)	PA5-85166, Invitrogen	1:200
**Anti-Vimentin Antibody from mouse**	Vimentin	V2258, Sigma-Aldrich	1:200
**Anti-Vimentin Antibody from mouse**	Vimentin	MAB1681, Sigma-Aldrich	1:200
**Alexa Fluor 594 Phalloidin**	F-actin	A12381, Invitrogen	1:400
**Rhodamine-Phalloidin**	F-actin	R415, Invitrogen	1:400
**Donkey anti-rabbit IgG (H+L), Alexa Fluor 647**	Rabbit IgG (secondary)	A31573, Invitrogen	1:200

**Goat anti-rabbit IgG (H+L), Alexa Fluor 568**	Rabbit IgG (secondary)	A-11036, Invitrogen	1:200
**Alexa Fluor 488 goat anti-rabbit IgG (H+L), highly cross-adsorbed**	Rabbit IgG (secondary)	A-11034, Invitrogen	1:200
**Goat anti-mouse IgG (H+L), Alexa Fluor Plus 647, highly cross-adsorbed**	Mouse IgG (secondary)	A32728, Invitrogen	1:200
**Goat anti-mouse IgG (H+L), Alexa Fluor Plus 647, highly cross-adsorbed**	Mouse IgG (secondary)	A32728, Invitrogen	1:200
**Goat anti-mouse IgG (H+L), Alexa Fluor Plus 647, highly cross-adsorbed**	Mouse IgG (secondary)	A32728, Invitrogen	1:200
**Alexa Fluor 488 goat anti-mouse IgG**	Mouse IgG (secondary)	A-11001, Invitrogen	1:200

Table S2. Gene expression assays

Gene name (aliases)	TaqMan assay ID	Supplier
**ALB (Albumin)**	Hs00609411_m1, FAM	Thermo Fisher Scientific
**ACTB (β-actin)**	Hs01060665_g1, FAM	Thermo Fisher Scientific
**COL1A1 (Collagen type I alpha 1 chain)**	Hs01103890_m1, FAM	Thermo Fisher Scientific
**MYOG (Myogenin)**	Hs01072232_m1, FAM	Thermo Fisher Scientific
**MYF5 (Myogenic factor 5)**	Hs00271574_m1, FAM	Thermo Fisher Scientific
**MYF6 (Myogenic factor 6, MRF4)**	Hs00231165_m1, FAM	Thermo Fisher Scientific
**MYH7 (Myosin heavy chain 7, β-MHC)**	Hs01110632_m1, FAM	Thermo Fisher Scientific
**MYOD1 (Myogenic differentiation 1)**	Hs00159528_m1, FAM	Thermo Fisher Scientific
**TRIM63 (Tripartite motif containing 63, MuRF1)**	Hs00822397_m1, FAM	Thermo Fisher Scientific
**FBXO32 (F-box protein 32, Atrogin-1)**	Hs01041408_m1, FAM	Thermo Fisher Scientific
**GAPDH (Glyceraldehyde-3-phosphate dehydrogenase)**	Hs02786624_g1, VIC	Thermo Fisher Scientific
**B2M (Beta-2-microglobulin)**	Hs00187842_m1, VIC	Thermo Fisher Scientific
**RPLP0 (Ribosomal protein lateral stalk subunit P0, also known as 36B4)**	Hs00420895_gH, VIC	Thermo Fisher Scientific
